# Dynamic Correlation Between Bacterial Communities and Volatile Compounds During Douchiba Fermentation

**DOI:** 10.1002/fsn3.70153

**Published:** 2025-04-07

**Authors:** Yurou Yang, Panpan Yang, Anyan Wen, Haiying Zeng, Na Liu, Likang Qin, Pengsen Zhou

**Affiliations:** ^1^ School of Liquor and Food Engineering Guizhou University Guiyang China; ^2^ Key Laboratory of Agricultural and Animal Products Storage and Processing of Guizhou Province Guiyang China; ^3^ National & Local Joint Engineering Research Center for the Exploitation of Homology Resources of Medicine and Food Guiyang China; ^4^ Qiongfang Food Development co., Ltd. Bijie China

**Keywords:** bacterial diversity, correlation analysis, Douchiba, volatile compounds

## Abstract

Douchiba is a traditional bacteria‐fermented soybean product in Guizhou, refined through the following steps of soaking, steaming, koji‐making, salt sprinkling, fermentation, pounding, drying, molding, and after‐ripening. However, the dynamic alterations of microbiota and volatile compounds of Douchiba were seldom reported. In this work, the bacterial communities and volatile compounds during the fermentation process of Douchiba were explored by high‐throughput sequencing and headspace solid‐phase microextraction and gas chromatography–mass spectrometry (HS‐SPME‐GC‐MS) techniques, respectively. Furthermore, the correlation between microbial communities and volatile compounds was analyzed with Spearman's method. The findings revealed that the diversity and evenness of bacteria increased during the fermentation process of Douchiba. The dominant bacteria genera included *Bacillus*, *Staphylococcus*, *Tetragenococcus*, *Loigolactobacillus*, and *Virgibacillus*. *Bacillus* dominated the bacterial communities throughout the fermentation process, and *Staphylococcus*, *Tetragenococcus*, *Loigolactobacillus*, and *Virgibacillus* also became the dominant genera in the fermentation and after‐ripening stages. A total of 134 volatiles were identified. The main volatiles were ketones and alcohols in the koji‐making stage, ketones and pyrazines in the fermentation stage, and acids in the after‐ripening stage. In total, 26 compounds were selected as the main characteristic volatiles, including 2,3,5‐trimethylpyrazine, 3‐methyl‐1‐butanol, and indole, which endowed Douchiba with a specific flavor. Additionally, *Bacillus* was significantly positively correlated with isovaleraldehyde, benzaldehyde, and acetic acid. *Staphylococcus* was significantly positively correlated with indole, 3‐methylbutanoic acid, and eucalyptol. *Tetragenococcus*, *Loigolactobacillus*, and *Virgibacillus* were significantly correlated with indole and estragole. The research establishes a theoretical foundation for better regulating the flavor quality of Douchiba products.

## Graphical abstract

In Douchiba 134, volatile compounds and 10 dominant bacteria were identified. The 26 characteristic volatiles work together to give Douchiba its unique flavor. The five key bacteria were positively related to most characterized volatiles.

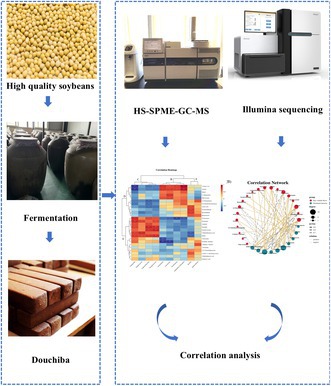

## 1 Introduction

Douchi is one of the four well‐known fermented soybean products in China (Douchi, soybean paste, soy sauce, and sufu) (Li et al. 2019; Que et al. 2023). Currently, on the basis of dominant fermentation microbes, Douchi can be categorized as *Mucor*‐type, *Aspergillus*‐type, *bacterial*‐type, and *Rhizopus*‐type. Douchiba is a derivative of *bacterial*‐type Douchi, such as Douchiba in Bijie, Guizhou. Compared with Douchi, Douchiba shows local differences in production technology and flavor. In the production process of Douchiba, with soybeans as the raw material, Douchi products are first obtained through soaking, steaming, koji‐making, salting, and fermentation, and then turned into the final product through mashing, drying, molding, and after‐ripening (Fan et al. 2019). Due to the special microbial flora and unique production process, Douchiba has unique quality characteristics, such as a unique flavor, a delicious taste, and a black appearance. Douchiba can be used to prepare dishes with various cooking methods so as to increase the color and aroma of foods (Zheng 2021). In addition, Douchiba has been used as the preferred raw material of related condiments in many chili companies.

Microorganisms and their associated enzymes largely determine the nutrients, flavor, and functional components of fermented soy products (Li et al. 2024; Li, Wang et al. 2024; Liu et al. 2024). For instance, it has been reported that *Lactobacillus* and *Tetragenococcus* in fermented soy sauce were respectively positively correlated with succinic acid and lactic acid (Feng et al. 2023). Similarly, it was found that *Enterobacter* and *Enterococcus* had a significant effect on the production of amino acids during the fermentation of sufu (Huang et al. 2018). However, the fermentation process of *bacterial*‐type Douchi involves many microbial species, mainly including *Bacillus*, *Micrococcus*, and *Lactobacillus* (Lu and Zheng 2011). *Bacillus* is the primary microbe responsible for the production of koji‐making and fermentation in *bacterial*‐type Douchi (Chen et al. 2022). The most common *B. subtilis natto* is significant in the synthesis of flavoring compounds such as amino acids and polypeptides. It is evident that the structure of microorganisms in fermented soybean products contributes significantly to the production of flavor and nutrition. So far, the formation and configuration of the bacterial community in the Douchiba fermentation process have seldom been reported. Therefore, it is necessary to explore the evolution of the bacterial community during the fermentation process of Douchiba.

Aroma is not only one of the important sensory characteristics to measure the quality of traditional seasonings, but also a major sensory factor that directly affects consumers' satisfaction and desire to purchase (Yu et al. 2018). During the fermentation process, Douchiba formed a unique aroma under the synergistic effect of microorganisms and their enzyme system. According to a study, the volatile compounds in Douchiba mainly included alcohols, acids, esters, and aldehydes and were rich in free amino acids, especially bitter amino acids (Qin and Ding 2007). In addition, Zhang et al. (2022) reported that major components of Ba‐bao Douchi (*bacterial*‐type) flavor include esters, aldehydes, and terpenes, and that the key compounds were positively correlated with *Acetobacter*, *Pseudomonas*, *Porphyrobacter*, *Arthrobacter*, and *Ralstonia*. The relationship between aroma and microbiota largely affects the selection of aroma‐related bacteria as the starters for the development of Douchiba products. However, the relationship between key volatile compounds and core strains of Douchiba during the fermentation process was seldom reported.

In this work, the samples of Douchiba were collected at various fermentation stages, and the bacterial communities in Douchiba were investigated with high‐throughput sequencing. Additionally, the volatile flavors in Douchiba were characterized with HS‐SPME‐GC‐MS. Meanwhile, orthogonal partial least squares‐discriminant analysis (OPLS‐DA) and relative odor activity value (ROAV) were combined to identify key volatile compounds during the fermentation of Douchiba. Finally, the correlation network analysis was performed to reveal the association of core genera with the key volatile compounds. The study aims to give a scientific rationale for the directed modulation of the flavor of Douchiba.

## 2 Materials and Methods

### 2.1 Preparation of Samples

**FIGURE 1:**
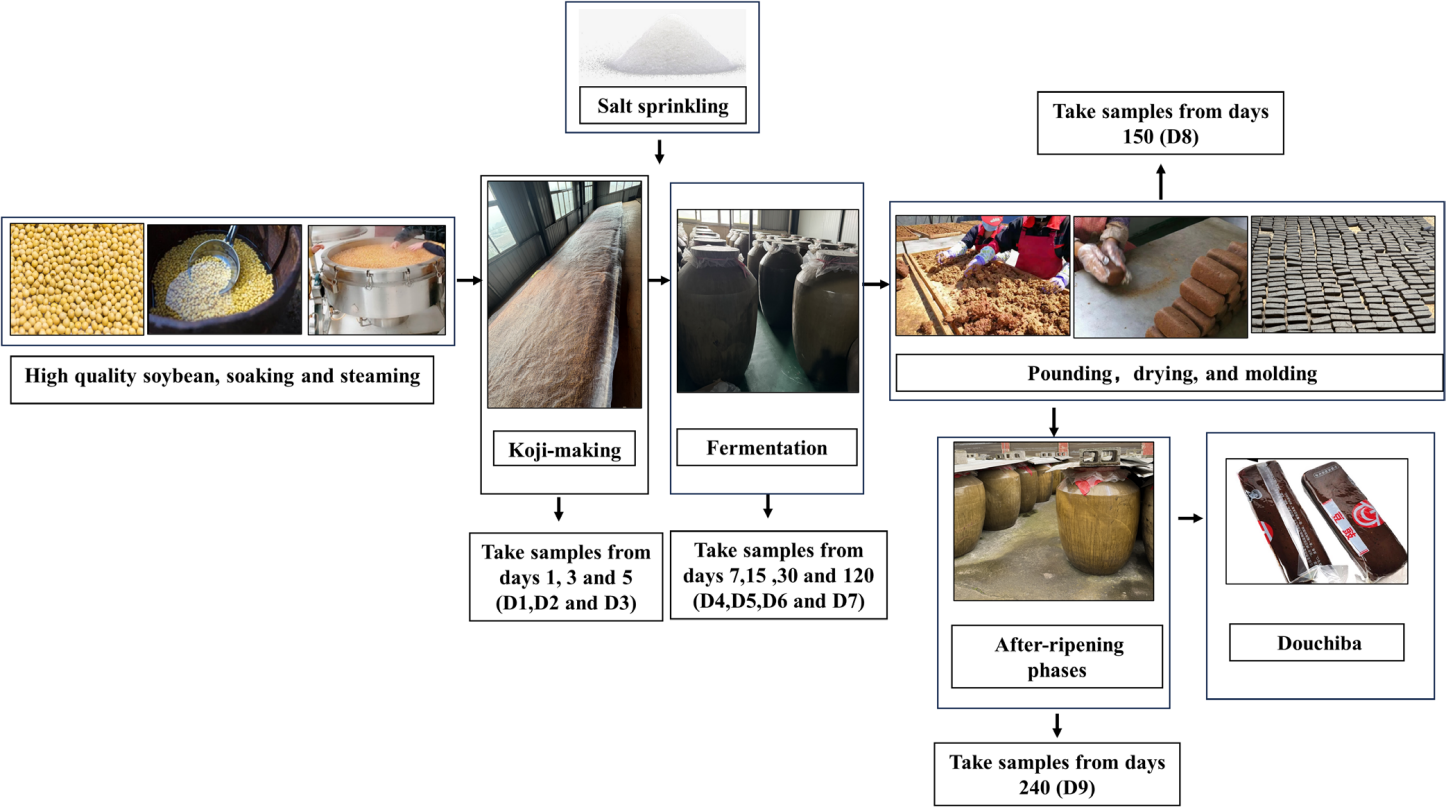
The production process of Douchiba.

The samples of traditional naturally fermented Douchiba were prepared by Qiongfang Food Development Co. Ltd. (Bijie, China). All samples were collected during the koji‐making stage (Days 1, 3, and 5), fermentation phase (Days 7, 15, 30 and 120), pounding, drying, and molding stages (Days 150) and maturation stage (Days 240) and three replicates were set (Figure 1). The samples were labeled D1 (Day 1), D2 (Day 3), D3 (Day 5), D4 (Day 7), D5 (Day 15), D6 (Day 30), D7 (Day 120), D8 (Day 150), and D9 (Day 240) and then preserved in the −80°C laboratory refrigerator for subsequent examination.

### 2.2 Determination of Bacterial Community

A sample (2 g) was added to the container and then entirely ground with liquid nitrogen. The sample (0.2 g) was then transferred to EP tubes for DNA extraction with the CTAB method. DNA concentration was diluted to 1 μg/μL with sterile water after DNA quality was assayed by 1% agarose gel electrophoresis. The V3‐V4 region of the bacterial 16S rRNA gene was amplified with primer pairs 341F (5′‐CCTAYGGGRBGCASCAG‐3′) and 806R (5′‐GGACTACNNGGGTATCTAAT‐3′) by a Bio‐rad T100 Gradient PCR Instrument (T100, USA). Following purification and construction of paired‐end libraries, sequencing of PCR amplification products was performed on the Illumina NovaSeq PE250 platform (Novogene Co. Ltd. Beijing, China). Using QIIME2 (v 2022.2) software to process high‐quality bacterial sequencing reads were processed, and noise reduction was performed with the DADA2 module to obtain final amplicon sequence variants (ASVs) and characterization tables. QIIME2 software was used for species annotation and rapid multiple sequence comparison, and the data were finally normalized for subsequent evaluation of the alpha diversity index and beta diversity analysis.

### 2.3 Extraction of Volatile Compounds (HS‐SPME)

A sample (5.0 g) was transferred into a 20‐mL headspace vial. The aged SPME fiber (50/30 μm CAR/PDMS/DVB) was inserted into the headspace portion of the vial for 30 min adsorption at 50°C. The adsorption head was then removed and inserted into the injection port of the gas chromatography for 3 min desorption at 250°C. Then, the instrument started to acquire the data. The C6–C26 n‐alkanes were analyzed under the same chromatographic conditions as the samples, and the retention index (RI) of substances was computed by the equipment operator software.

### 2.4 GC–MS Analysis of Volatile Compounds

GC analysis was performed under the following conditions: DB‐Wax column (30 m × 0.25 mm, 0.25 μm, Agilent, USA), injection temperature (250°C), and high‐purity He as the carrier gas (1.0 mL/min). In the gradient program, the column temperature was set at 40°C, maintained for 3 min, then increased to 230°C (10°C/min) and maintained for 6 min.

MS conditions were set as follows: interface temperature (250°C), electron capacity in EI mode (70 eV), detector voltage (0.2 kV), and ion source temperature (200°C).

Qualitative and quantitative analyses were performed as follows. The information retrieved from the mass spectral library (NIST 17) was aligned and then the retention index (RI) was calculated. Then, based on the mass spectra, the compounds were determined and the compounds with similarity (SI) > 800 were selected. The relative contents were calculated using the peak area normalization method (Wang, Wen, et al. 2023; Wang, Lei et al. 2023). RI was calculated as follows (Chen et al. 2024):

(1) $$\mathrm{RI}=10\text{0n}+\frac{t_{i}-t_{n}}{t_{n+1}+t_{n}}\times 100,$$

where *t*_i_ (min) is the retention time of the component to be analyzed; *t*_n_ (min) is the retention time of n‐alkanes with n carbon atoms; *t*_n+1_ (min) is the retention time of n‐alkanes with *n* + 1 carbon atoms (*t*_n+1_ > *t*_i_>*t*_n_).

### 2.5 ROAV Analysis

ROAV was used to screen the key volatile compounds in the fermentation process of Douchiba. The compound that contributed the most to the overall flavor in the sample was selected and recorded as ROAV_stan_ = 100. ROAV of other volatile compounds in the sample was calculated as (Miao et al. 2024):

(2) $$\text{ROAV}=\frac{C_{i}}{C_{\text{stan}}}\times \frac{T_{\text{stan}}}{T_{i}}\times 100,$$

where *C*_i_ (%) and *T*_i_ (μg/kg) respectively represent the relative content and sensory threshold of volatile compounds in Douchiba; *C*_stan_ (%) and *T*_stan_ (μg/kg) represent the relative content and sensory threshold of the most important volatile compound, respectively.

### 2.6 Statistical Analysis

The data from the experiments were presented as the average of three replicates. Species annotation and α‐ and β‐diversity analyses were performed with QIIME2 (v 2022.2) software. The linear discriminant analysis effect size (LEfSe) tool had been used to characterize changes in species abundance between samples. Histogram visualization of the relative abundance of superior bacterial genus and clustered heatmaps of flavors were plotted with Origin 2018 (v 9.50.00). With SIMCA (v 14.1.0.2047) software, the variable importance for the projection (VIP) from OPLS‐DA modeling was examined and then combined with ROAV to screen key volatile compounds. With SPSS (v 26.0.0.0), Spearman correlation coefficients (r) were computed, and *p* < 0.05 and |r| > 0.6 were considered as the significant correlation. Correlations between core bacterial species and key volatile compounds were estimated using omicstudio online (https://www.omicstudio.cn/tool).

## 3 Results and Discussion

### 3.1 Characterization and Dynamic Changes of the Bacterial Community of Douchiba

#### 3.1.1 Bacterial Community Analysis

**FIGURE 2:**
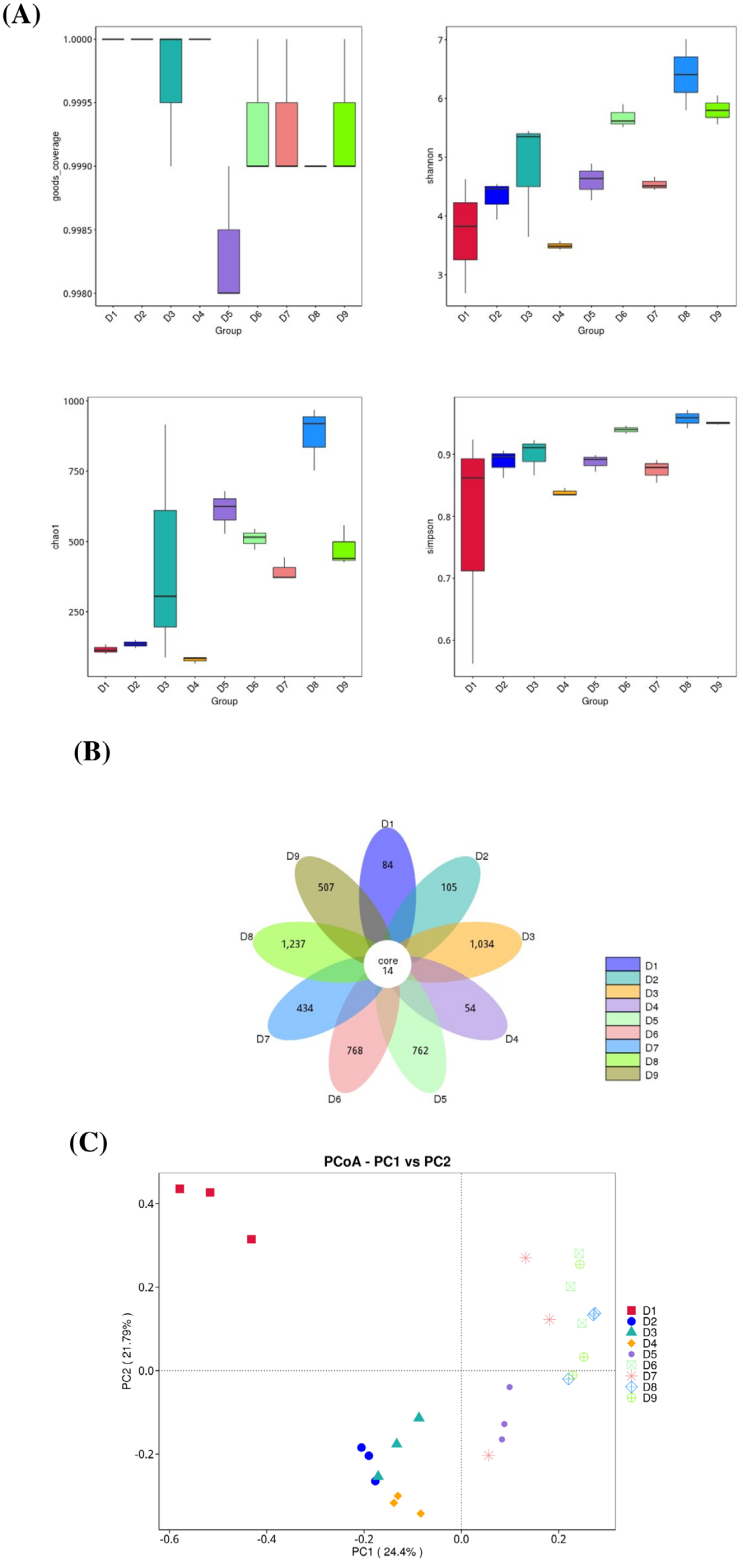
Changes in microbial community in fermented Douchiba during natural fermentation. (A) α‐diversity, (B) petal plots, and (C) β‐diversity analysis.

The variation of microbial diversity of Douchiba throughout the natural fermentation process was analyzed based on the α‐ and β‐diversity of microorganisms. The coverage indices of Douchiba samples in different fermentation stages were above 0.998 (Figure 2A), indicating the high accuracy and completeness of the sequencing data. The Chao1 index of the Douchiba samples showed an overall increasing trend throughout the fermentation process, revealing that the abundance of the microbial community gradually increased. The microbial abundance was lowest in D4. Shannon and Simpson indicators could be used to measure microbial diversity. Both the Shannon indicator and the Simpson indicator showed an absolute increasing trend throughout the fermentation process, showing an increase in the diversity of the bacterial community during the Douchiba fermentation process. The petal plots (Figure 2B) showed that there were 14 shared ASVs in nine fermentation stages from D1 to D9. The number of unique ASVs gradually increased during fermentation. In summary, the richness and variety of the bacteria in Douchiba changed significantly throughout the fermentation process. It might be interpreted as follows: The environmental factors such as fermentation acidity and nutrient conditions changed, and the competitive relationship among microorganisms existed.

The β‐diversity of nine groups of samples was analyzed by PcoA to explore differences in microbial communities between samples (Figure 2C). The PcoA results showed that the samples at stage D1 were located in the second quadrant. The samples at stages D2–D4 were located in the third quadrant. The samples at stages D5–D9 were located in the first and fourth quadrants. The above results indicated that the bacterial community structure of Douchiba had a clear temporal succession pattern with fermentation time. During the koji‐making phase, the high‐water activity and nutrient abundance of cooked soybeans facilitated the growth of diverse microorganisms and thus led to substantial alterations in the bacterial microbiota (Suo et al. 2015). As fermentation proceeded, the proportion of bacterial species in Douchiba changed again due to the influences of temperature, humidity, drying, and molding. However, some bacteria, such as *Bacillus* and *Staphylococcus*, were highly resistant to the changes in the fermentation environment and thus their abundance in the post‐fermentation stage was higher (Rhee et al. 2011). These bacteria might contribute to the similarity of bacterial communities in Douchiba samples in stages D5–D9. In summary, the bacterial community structure of Douchiba changed significantly at different fermentation stages. This finding was close to that of Zhang et al. (2022).

#### 3.1.2 Microbial Composition and Dynamics Analysis

**FIGURE 3:**
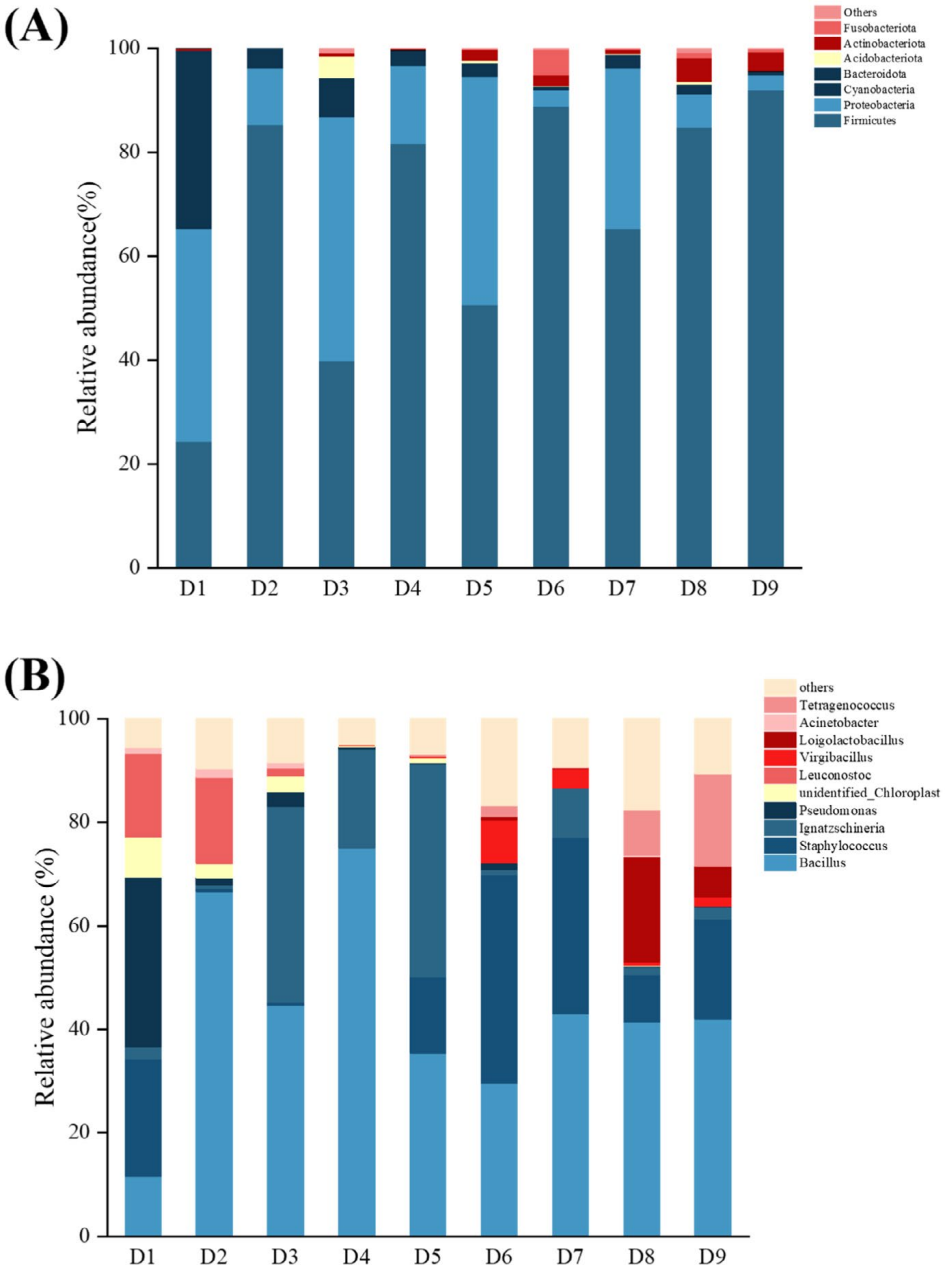
Relative abundance of bacterial composition. (A) At the phylum level and (B) at the genus level.

Figure 3 shows the community structure and species abundance at the phylum and genus level during fermentation of Douchiba. At the phylum level (Figure 3A), the bacterial community of Douchiba included a total of seven phyla (relative abundance > 1%): Firmicutes, Proteobacteria, Cyanobacteria, Bacteroidota, Acidobacteriota, Actinobacteriota, and Fusobacteriota. Among them, Firmicutes, Proteobacteria, and Cyanobacteria were the dominant phyla in D1, and the relative abundance reached 99.36%. During fermentation, the abundance of Firmicutes showed a fluctuating upward trend until the end of fermentation (D9) when the final abundance reached 91.91%. Firmicutes was the dominant phylum during fermentation. Similar results were reported in the study on *Aspergillus*‐type Douchi (Zhang, Li, et al. 2022; Zhang, Lin, et al. 2022). The average relative abundance of Proteobacteria was 22.33%. Its abundance fluctuated from 3.12% to 46.96% from D1 to D7 and gradually decreased to 2.86% in the subsequent phases. Compared to the abundances of Firmicutes and Proteobacteria, the relative abundance of Cyanobacteria reached 34.12% in D1 and became low (< 1%) in later fermentation stages.

At the genus level, the top 10 core genera in terms of relative abundance included *Bacillus*, *Staphylococcus*, *Ignatzschineria*, *Pseudomonas*, *unidentified_Chloroplast*, *Leuconostoc*, *Virgibacillus*, *Loigolactobacillus*, *Acinetobacter*, and *Tetragenococcus*. The relative abundances of superior genera in the Douchiba samples varied a across fermentation phases (Figure 3B). The average relative abundance of *Bacillus* was 43.31%, which dominated the whole fermentation stage. According to the earlier study (Li et al. 2017), *Bacillus* might quicken the fermentation process of Douchi and supply the nutrients needed for additional bacteria. Furthermore, the abundance of other dominant bacteria changed remarkably during the fermentation process. It was noted that *Staphylococcus*, *Tetragenococcus*, *Loigolactobacillus*, or *Virgibacillus* were not the dominant genera from D1 to D4. However, they gradually became the dominant genera, and their relative abundances increased significantly from D5 to D9. In D9, the abundances of *Staphylococcus*, *Tetragenococcus*, *Loigolactobacillus*, and *Virgibacillus* were 19.43%, 17.89%, 5.85%, and 1.77%, respectively. At the end of fermentation, these genera became the dominant bacteria and contributed to the ultimate flavor and quality formation. It was reported in an earlier study that *Staphylococcus* was well adapted to fermented Douchi and involved in ester production along with *Bacillus* (Furuhata et al. 2010). *Tetragenococcus* is commonly found in fermented foods, and *Tetragenococcus halophilus* , as the most common species in *Tetragenococcus*, could increase the contents of organic acids, aldehydes, esters, and other flavor substances (Cui et al. 2014). *Loigolactobacillus* was identified as a dominant genus in salt‐free fermented vegetables (Liu et al. 2023). *Virgibacillus* belongs to the order of *Bacillus* and is a moderately halophilic bacterium with high protease production capacity (Sinsuwan et al. 2012). Nonetheless, the relative abundances of other bacterial genera such as *Ignatzschineria*, *Pseudomonas*, *unidentified‐chloroplast*, *Leuconostoc*, and *Acinetobacter* decreased in the later fermentation process. The declines in the relative abundances of these dominant strains might be related to the antimicrobial metabolites generated by the dominant strains and the competitive utilization of nutrients with other species during fermentation (Xiao et al. 2020).

**FIGURE 4:**
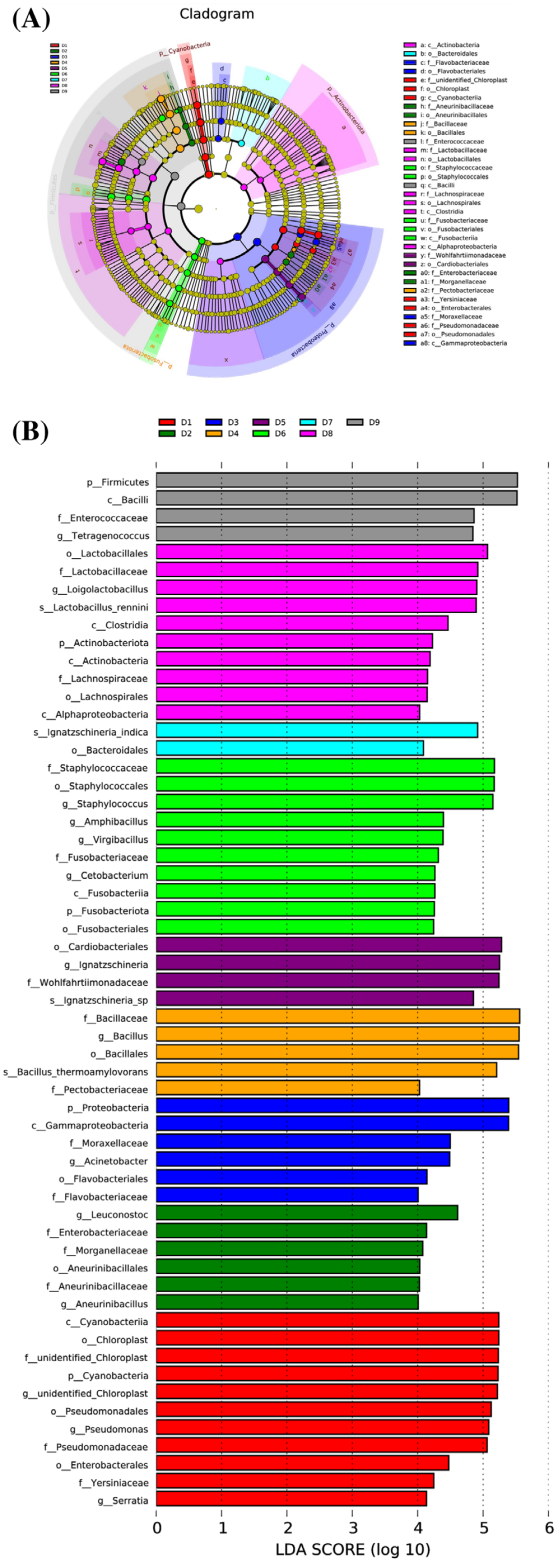
LEfSe analysis of microbial communities. (A) LEfSe taxonomic cladogram and (B) differential species score.

Through linear discriminant analysis effect size (LEfSe) analysis, key microorganisms with statistical differences between various fermentation samples could be identified. On the genus level, a sum of 13 bacterial genera with significant differences were detected among Douchiba samples (Figure 4). The biomarkers in different stages were identified as follows: *Serratia*, *Pseudomonas*, and *unidentified_Chloroplast* in D1; *Leuconostoc* in D2; *Acinetobacter* in D3; *Bacillus* in D4; *Ignatzschineria* in D5; *Cetobacterium*, *Virgibacillus*, *Enterococcus*, and *Staphylococcus* in D6; *Loigolactobacillus* in D8; and *Tetragenococcus* in D9. The distance between the samples in the D7 group was longer than that between the samples in other groups; no biomarker at the genus level was discovered in D7. Consequently, the genus with substantial differences was not found in the D7 group by LEfSe analysis.

### 3.2 Dynamics of Volatile Compounds During Douchiba Fermentation

**FIGURE 5:**
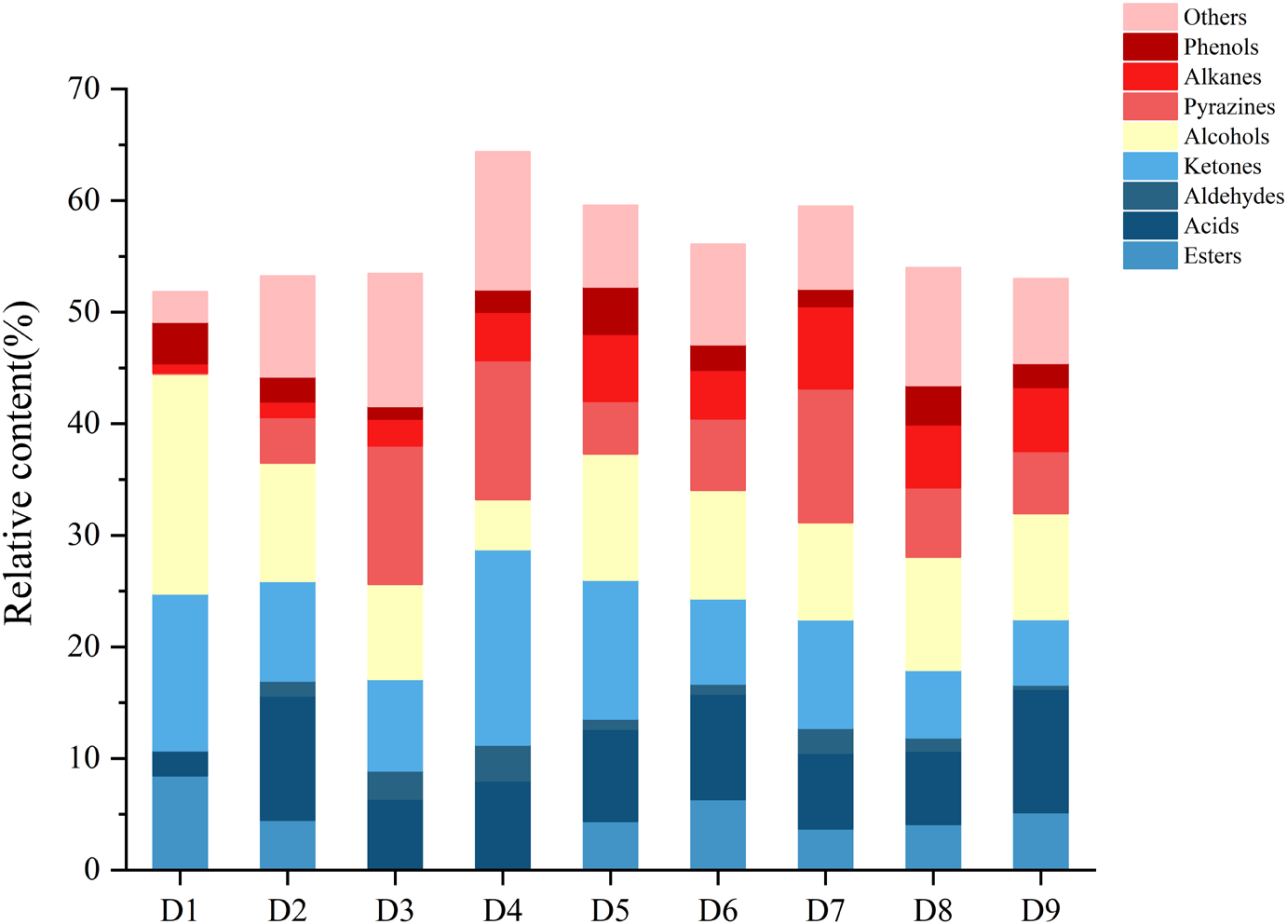
Changes in the relative contents of flavor substances in Douchiba at different fermentation stages.

A total of 134 volatiles were identified in different Douchiba samples by HS‐SPME‐GC–MS, including 14 esters, 16 acids, 3 aldehydes, 27 ketones, 18 alcohols, 9 pyrazines, 17 alkanes, 8 phenols, and 22 other substances (Table S1). Among these compounds, alcohols had the highest relative abundances (4.47%–19.64%) in Douchiba during fermentation, followed by ketones (5.89%–17.53%), pyrazines (0.14%–12.47%), and acids (2.17%–11.14%), indicating that alcohols, ketones, pyrazines, and acids were important volatile compounds in the fermentation process of Douchiba. The volatile flavor components of Douchiba showed significant differences during fermentation (Figure 5), as indicated by the increased relative contents of acids, aldehydes, pyrazines, and alkanes.

Alcohols are related to lipid oxidation, amino acid decomposition, methyl ketone reduction, and acid degradation, in which a typical sauce flavor of soybean products is formed (Ma et al. 2023). The relative contents of 1‐octen‐3‐ol and 1‐hexanol peaked in D1 and respectively reached 5.62% and 1.16%. 1‐Hexanol and 1‐octen‐3‐ol originated from boiled soybeans and gradually decreased during fermentation. Under the catalytic action of lipoxygenase, linoleic acid is converted into hydroperoxides, which are subsequently dehydrogenated into ketones and finally transformed into 1‐octen‐3‐ol (Yang et al. 2021). 3‐Methyl‐1‐butanol was generated from leucine via the Ehrlich degradation pathway in yeast (Connor and Liao 2008) and its relative content reached a maximum value of 4.84% in the D5 stage. However, its relative content gradually decreased in the later fermentation stages probably due to the depletion of precursors. It was speculated that the traditionally fermented Douchiba had the highest content of alcohols due to its long fermentation period and low temperature, which promoted the fermentation of salt‐tolerant aromatic yeasts (Wah et al. 2013).

Ketones are typically formed due to amino acid decomposition or microbial metabolism during fermentation and endow fermentation products with a distinct fruity aroma and scent (Hu et al. 2021). The relative content of 2‐butanone firstly rose, reached a maximum relative content of 2.54% in D4 and then decreased. The content of acetoin with a buttery and overripe fruity odor was initially high in Douchiba and gradually decreased from 6.83% to 0.21%. Acetoin had a low odor threshold and significantly increased the flavor even at low concentrations. *Bacillus subtilis* produced α‐acetolactic acid from sugar under aerobic circumstances, and subsequently α‐acetolactic acid was produced to acetoin catalyzed by α‐acetolactate decarboxylase produced by *Bacillus subtilis* (Liu et al. 2018).

Pyrazines are produced by the condensation of aminoketones in the Maillard reaction and Strecker degradation or microbial metabolism during fermentation (Chen et al. 2011). Pyrazines present nutty, chocolatey, or toast‐like aromas that endow Douchi with a characteristic flavor. In addition, pyrazines change the color of Douchi and enhance its flavor. Abundant pyrazines such as 2,5‐dimethylpyrazine (0.06% to 2.63%), 2‐ethyl‐3,5,6‐trimethylpyrazine (0.13% to 1.67%), 2,3,5,6‐tetramethylpyrazine (0.24% to 3.42%), and 2,3,5‐trimethylpyrazine (0.08% to 4.47%) were detected from stages D3 to D7. However, an increase in temperature from stages D8 to D9 resulted in a drop in the relative content of pyrazines (due to their volatility) and their transformation by microorganisms (Chen et al. 2023).

Acids are produced primarily by lipid oxidation and phospholipid and triglyceride hydrolysis and have an irritating odor. During Douchiba fermentation, a high content of acetic acid was generated (0.40%–5.35%). The sour taste in soy‐fermented foods was mainly ascribed to acetic acid produced by lactic acid bacteria via various metabolic pathways, such as pyruvate metabolism (An et al. 2021; Diez‐Simon et al. 2020). 3‐Methylbutanoic acid (0.14%–3.19%) and 2‐methylbutyric acid (0.46%–7.65%) were converted from branched‐chain amino acids via the Ehrlich pathway during fermentation (Feng et al. 2013). It was found that 2‐methylbutyric acid was the main aromatic compound in fermented soy products and generated a pungent acidic flavor. The relative content of isobutyric acid gradually increased and reached a maximum value of 2.33% during stage D4 but showed a fluctuating downward trend during the subsequent stages. It was speculated that some microorganisms used the nutrients in Douchiba to gradually produce isobutyric acid during the early stages of fermentation. As fermentation proceeded, isobutyric acid might have undergone esterification with alcohols, thus leading to a decrease in its content (Zhang et al. 2020).

Esters are usually formed in the esterification of alcohols and acids and endow products with a special fruity and floral flavor, so that they can also mask the unpleasant and irritating odor of free fatty acids (Flores et al. 2004). After 7 days of fermentation (D4), esters such as isoamyl acetate, isoamyl isobutyrate, 3‐methylbutyl‐2‐methyl butanoate, isoamyl isovalerate, and 2‐methoxyethyl acetate were produced largely and had a significant effect on the flavor of Douchiba. The increase in esters induced by the fermentation process should contribute to the characteristic flavor of Douchiba.

Along with the volatile compounds listed above, some volatile compounds such as N, N‐dimethylmethanamine, maltol, guaiacol, indole, β‐myrcene, and d‐limonene were also identified during the fermentation process of Douchiba. Among them, N, N‐dimethylmethanamine presented a fishy odor, and its relative content reached a maximum value (1.51%) at stage D4. Then, the relative content decreased sharply in the subsequent fermentation. It was hypothesized that its decrease might be ascribed to the metabolism of microorganisms such as *Lactobacillus* and *Enterococcus* (Park et al. 2020). Maltol (sweet flavor) and guaiacol (smoky flavor) were detected during the entire fermentation process and might play an important role in the flavor of Douchiba. Indole was considered to be the primary disagreeable flavor of stinky tofu (Tang et al. 2022). Therefore, it was hypothesized that indole also contributed to the unique flavor of Douchiba. In addition, β‐myrcene and d‐limonene were mainly derived from spice substances added during the fermentation of Douchiba (Ma et al. 2023).

### 3.3 Examination of the Distinctive Characteristics of Flavor in Douchiba

#### 3.3.1 Analysis of Differential Volatile Compounds Based on OPLS‐DA

**FIGURE 6:**
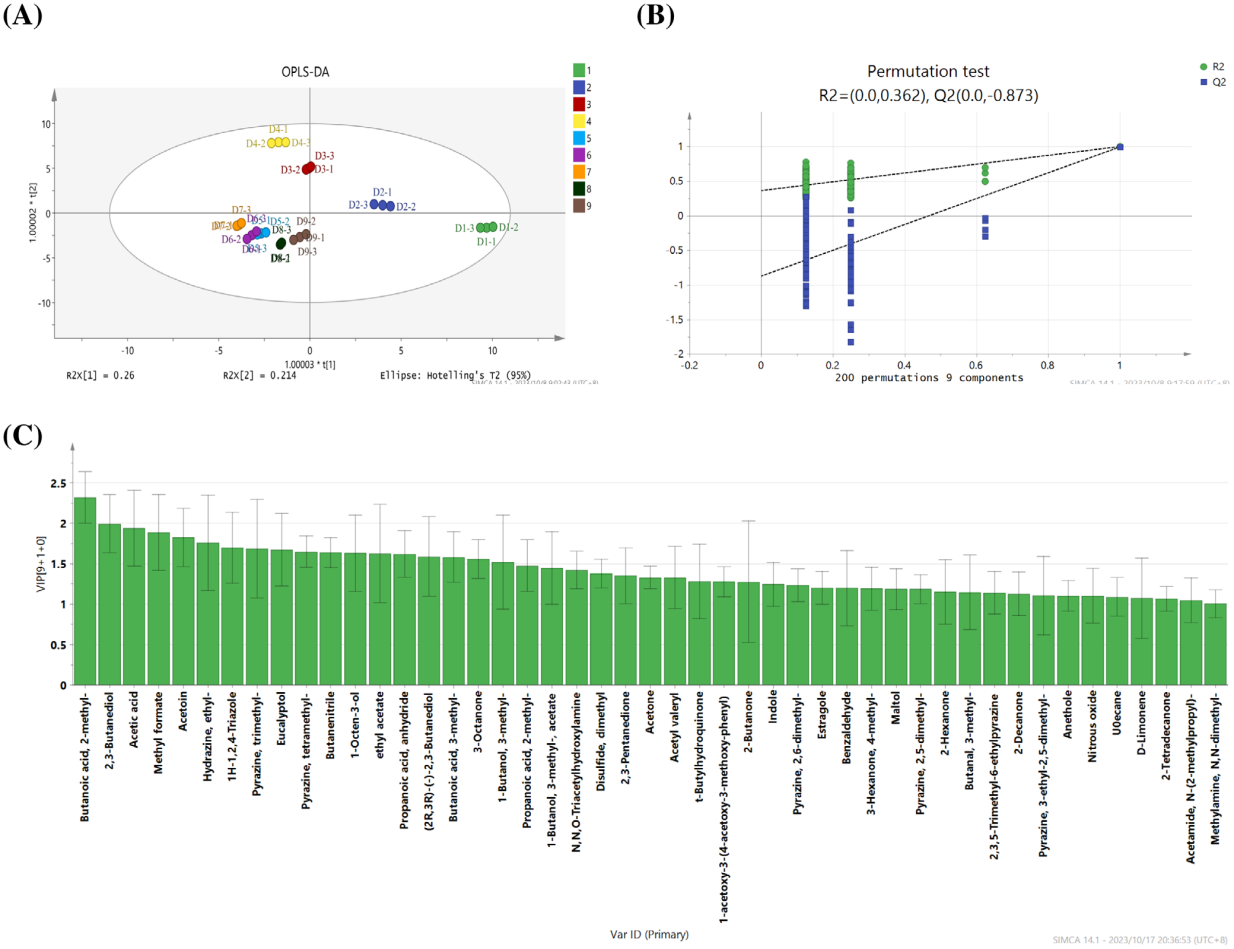
OPLS‐DA analysis of Douchiba during different fermentation periods. (A) OPLS‐DA plot of the samples, (B) Performance of the permutation test, and (C) compounds with VIP > 1.

OPLS‐DA is a suitable analytical tool for the categorization of the data with multicollinear and noisy variables. OPLS‐DA was used to examine the differences in flavor substances during fermentation of Douchiba in this study. In the calculation of the sample dataset, the cumulative R^2^X, R^2^Y, and Q^2^ values were respectively 0.973, 0.994, and 0.978, indicating that the model had good predictability and accuracy. In addition, except that the samples of Douchiba in D5 and D6 stages showed the partial overlap, the samples of Douchiba in other stages could be obviously distinguished (Figure 6A), indicating that the volatile components of Douchiba in different fermentation stages were significantly different.

To investigate the above differences, a permutation test (*n* = 200) was conducted to identify differences in the characteristics between Douchiba samples and to ascertain whether the model was overfitted. The model was subjected to 200 permutation tests (Figure 6B) and the intercepts of R^2^ and Q^2^ were respectively 0.362 and −0.873, demonstrating that the model was well fitted.

Compound importance was assessed with variable importance in projection (VIP). The compounds with VIP > 1 were selected as the important contributors to the differences among samples. Through statistical analysis, a total of 47 flavor substances were screened, including 4 acids, 2 aldehydes, 3 esters, 11 ketones, 5 alcohols, 6 pyrazines, 3 alkanes, 2 phenols, and 11 other compounds (Figure 6C). These characteristic substances might be responsible for the flavor differences in Douchiba during fermentation.

#### 3.3.2 Analysis of Key Volatile Compounds in Douchiba Fermentation Process

The contribution of different volatiles to the overall flavor of Douchiba is related to their relative content and depends on their odor thresholds (Zhang et al. 2023). ROAV can be used to assess the contribution to the overall aroma of samples from various volatile compounds. This method has been successfully applied to screen key aroma compounds in various fermented soybean products, such as Douchi (Miao et al. 2024), soy sauce (Tan et al. 2024), and gray sufu (Tian et al. 2023). In general, volatile compounds with ROAV ≥ 1 contribute significantly to the overall flavor, and the higher the ROAV value, the greater the contribution to the flavor of the samples. Building upon this theoretical framework, we implemented systematic ROAV analysis to identify determinant aroma compounds throughout the fermentation process of Douchiba.

In total, 28 kinds of volatile compounds with ROAV ≥ 1 were identified in the samples of Douchiba, including 3 esters, 4 acids, 2 aldehydes, 4 ketones, 4 alcohols, 2 pyrazines, 2 alkanes, 2 phenols, and 5 others (Table S2). Based on the standard of ROAV ≥ 1 and VIP > 1, 26 volatile flavor substances were further screened as key flavor substances in the fermentation process of Douchiba, including 1‐octen‐3‐ol, 3‐octanone, and maltol.

**FIGURE 7:**
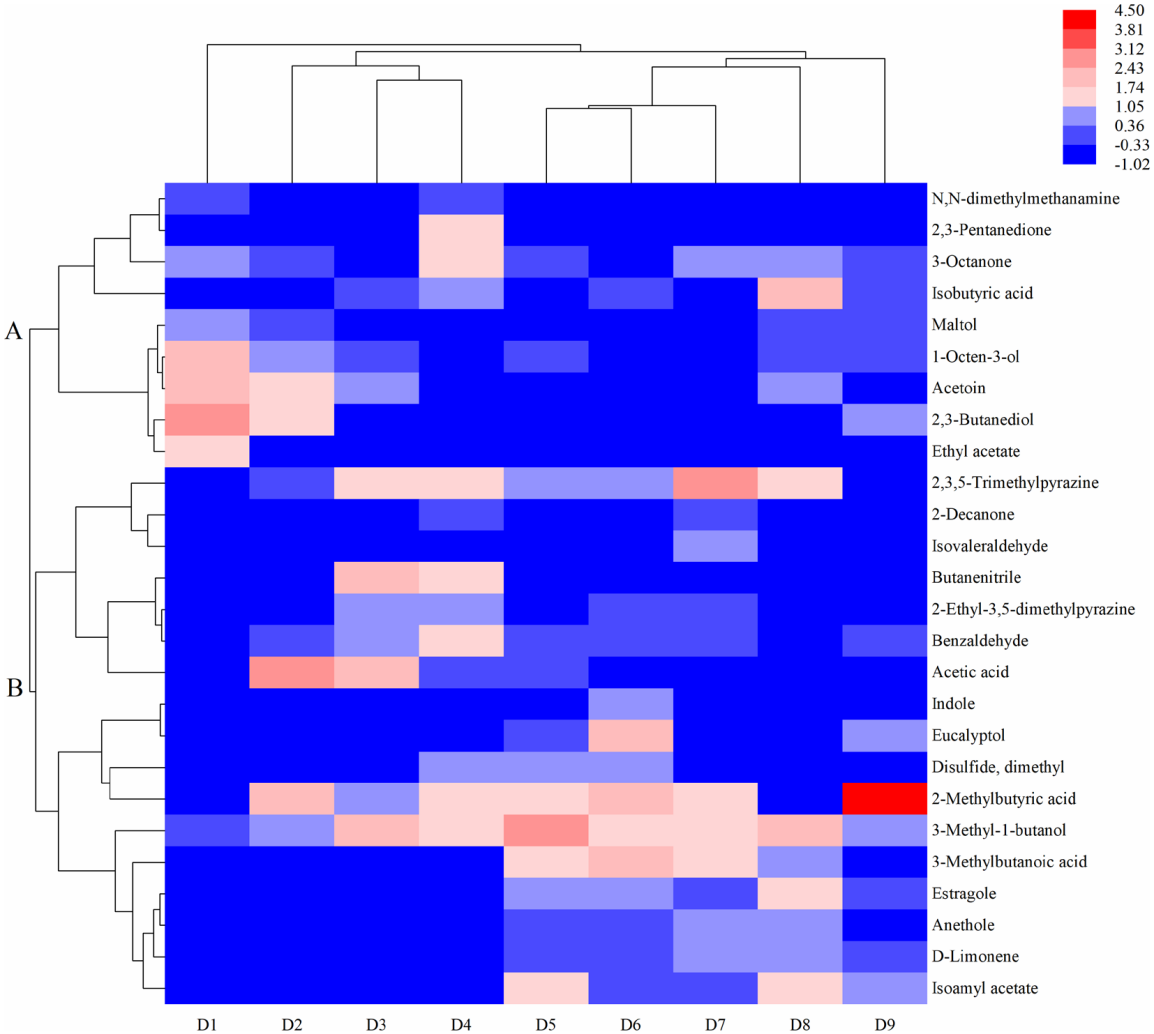
Heatmap visualization of key volatile compounds in Douchiba at different fermentation stages.

Heatmap cluster analysis was performed to further reveal the differences in the aroma composition among Douchiba samples at different fermentation stages (Figure 7). The relative contents of the 26 characteristic volatiles were set as variables, and each variable was normalized by summation. The normalized color intensity scale represented the low‐to‐high abundance of volatiles, and the maximum and minimum abundances were respectively shown in red and blue. In addition, volatile compounds could be categorized into two groups. Group A mainly consisted of alcohols and ketones, whereas Group B contained mainly acids, alcohols, and pyrazines. At stages D1–D4, five volatiles (2,3‐butanediol, ethyl acetate, acetoin, 1‐octen‐3‐ol, and 3‐methyl‐1‐butanol) contributed more to the aroma of Douchiba. Chen et al. (2021) found that 1‐octen‐3‐ol formed the mushroom aroma of Douchi fermented for 3–9 days, similar to the experimental results in the study. In addition, 1‐Octen‐3‐ol enhanced the aroma of the remaining aroma substances (Li, Wang et al. 2024; Li et al. 2024). 3‐Methyl‐1‐butanol and isoamyl acetate endowed Douchiba with a prominent fruity flavor (Wang et al. 2024). The volatiles contributing to the aroma of Douchiba at stages D5–D9 included 2‐methylbutyric acid, 2,3,5‐trimethylpyrazine, estragole, and 3‐methylbutyric acid. Wang, Lei et al. (2023) showed that 2,3,5‐trimethylpyrazine and indole contributed significantly to the aroma of mature Douchiba, which is similar to our study.

### 3.4 Correlation of Bacterial Genera With Characteristic Volatile Compounds

**FIGURE 8:**
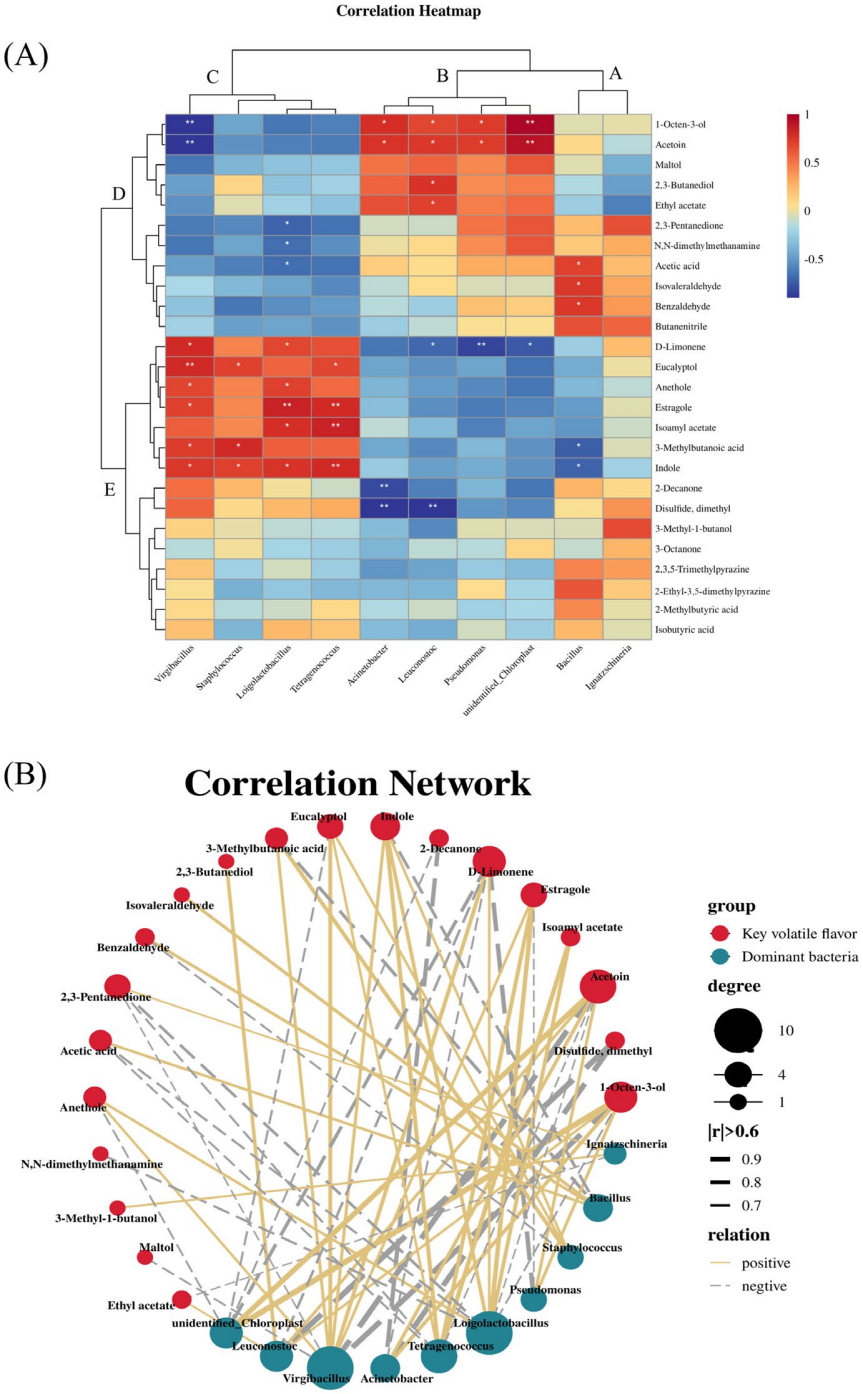
Relationships between dominant bacteria and 26 key volatile compounds visualized in a correlation clustering heat map (A) and correlation network (B), based on the relative content of key volatile compounds and the relative abundance of dominant bacteria.

The correlation between bacterial communities and key volatile compounds was investigated by spearman correlation analysis. The 10 predominant bacterial genera (Figure 8A) were grouped into 3 categories (A, B, and C) and the characteristic volatile compounds were grouped into 2 categories (D and E). Volatile compounds of Type D were positively correlated with the microbial genera of Types A and B and negatively correlated with the microbial genera of Type C. The volatile compounds of Type E were positively correlated with the genera of Type C and negatively t correlated with most genera of Types A and B. However, whether these microorganisms produced and absorbed relevant volatile compounds remained to be further investigated.

The correlation networks between 10 dominant genera and 26 characteristic volatile compounds were constructed, and the correlations were visualized in Figure 8B. Ten dominant genera were correlated with 20 key volatile compounds (|r| > 0.6). Five bacterial genera (*Bacillus*, *Staphylococcus*, *Tetragenococcus*, *Loigolactobacillus*, and *Virgibacillus*) showed significant positive correlation with 10 key volatile compounds (|r| > 0.6, *p* < 0.05).

*Bacillus* was significantly positively correlated with isovaleraldehyde, benzaldehyde, and acetic acid (Figure 8B). *Bacillus* produced proteases, amylase, pyrazine, and organic acids, which played a significant role in flavor formation (Xi et al. 2022). *Staphylococcus* showed a significant positive correlation with indole, 3‐methylbutanoic acid, and eucalyptol. This correlation might be interpreted as follows: *Staphylococcus* was the dominant genus in many fermented meats and had excellent metabolic ability for proteins and fats. *Staphylococcus* contributes to the production of short‐chain aldehydes, esters, and alcohols during the fermentation process, and the resulting flavor substances could be converted into complex volatile substances in the fermentation environment (Ma et al. 2023). *Staphylococcus* has been found to be positively associated with esters and alcohols, such as ethyl acetate and ethanol (Wang et al. 2021). *Tetragenococcus* showed a significant positive correlation with four volatile compounds, including isoamyl acetate, eucalyptol, indole, and estragole. *Tetragenococcus* widely exists in salt‐containing fermented foods and belongs to *Lactobacillus acidophilus* . Moreover, *Tetragenococcus* was reported to participate in the synthesis of amino acids and the production of volatile flavor substances such as aldehydes, alcohols, ketones, and esters, which enhanced the flavor of fermented foods (He et al. 2016). *Loigolactobacillus* and *Virgibacillus* were positively correlated with d‐limonene and indole. In addition, other microbial genera, such as *Pseudomonas*, *unidentified_Chloroplast*, *Leuconostoc*, and *Acinetobacter*, were positively correlated with some compounds, including 1‐octen‐3‐ol and acetoin. Similar to *Lactobacillus*, *Leuconostoc* was also a probiotic genus that increased the contents of certain acids and alcohols. In the after‐ripening stages, the growth of the above genera was hindered, and their abundance was extremely low. These genera had no significant influence on the mature flavor of Douchiba.

In short, *Bacillus*, *Staphylococcus*, *Tetragenococcus*, *Loigolactobacillus*, and *Virgibacillus* were the core bacterial genera responsible for the formation of characteristic volatile compounds during Douchiba fermentation. Nevertheless, the existence of these genera did not necessarily mean that these volatiles were produced. The role of these genera in the formation of the flavor of Douchiba required further exploration through inoculation experiments.

## 4 Conclusion

In this study, the changes in bacterial communities and volatile compounds during fermentation of Douchiba were studied. In addition, core microorganisms and key volatile compounds were identified and the correlations between them were explored. The five genera (*Bacillus*, *Staphylococcus*, *Tetragenococcus*, *Loigolactobacillus*, and *Virgibacillus*) were the dominant genera of Douchiba. A total of 134 volatiles were identified. In total, 26 volatiles were considered as key volatiles, including 1‐octen‐3‐ol, 2,3,5‐trimethylpyrazine, 3‐methyl‐1‐butanol, and indole. The correlation analysis showed that the five core bacterial genera were significantly positively correlated with most of the compounds, which performed an essential function in the formation of the flavor of Douchiba. The study improved the comprehension about the core flavor‐producing microorganisms in the fermentation process of Douchiba and provided the theoretical basis for screening bacterial leavener. Further integration of metabolomics and transcriptomics would reveal the metabolic pathways involved in the production of key flavor compounds in core functional microorganisms.

## Author Contributions

**Yurou Yang:** formal analysis (equal), methodology (equal), writing – original draft (equal), writing – review and editing (equal). **Panpan Yang:** formal analysis (equal), investigation (equal), resources (equal), supervision (equal). **Anyan Wen:** methodology (equal), project administration (equal), supervision (equal), writing – review and editing (equal). **Haiying Zeng:** funding acquisition (equal), project administration (equal), supervision (equal). **Na Liu:** conceptualization (equal), methodology (equal), supervision (equal). **Likang Qin:** funding acquisition (equal), project administration (equal), resources (equal), supervision (equal). **Pengsen Zhou:** project administration (equal), resources (equal).

## Conflicts of Interest

The authors declare no conflicts of interest.

## Supporting information


Data S1.

## Data Availability

Data is available on request.
