# Supplementary material for: Dynamic Correlation Between Bacterial Communities and Volatile Compounds During Douchiba Fermentation
Source: Food Sci Nutr. 2025 Apr 7;13(4):e70153. doi: 10.1002/fsn3.70153 (PMC11975051; doi:10.1002/fsn3.70153)
Supplement: Supplementary file 1 — Data S1. [file FSN3-13-e70153-s001.docx]

**Tables**

Table S1

Relative content (%) of volatile compounds in different fermentation stages by HS-SPME-GC–MS.

| **NO.** | **Compounds name** | **D1** | **D2** | **D3** | **D4** | **D5** | **D6** | **D7** | **D8** | **D9** |
| --- | --- | --- | --- | --- | --- | --- | --- | --- | --- | --- |
|  | **Esters (14)** |  |  |  |  |  |  |  |  |  |
| V1 | Ethyl isovalerate | 1.17 ± 0.27^a^ | ND | ND | ND | 0.15 ± 0.05^b^ | 0.08 ± 0.02^b^ | 0.10 ± 0.02^b^ | 0.25 ± 0.06^b^ | 0.08 ± 0.01^b^ |
| V2 | Methyl isovalerate | 0.87 ± 0.13^a^ | ND | ND | 0.02 ± 0.01^d^ | ND | 0.59 ± 0.03^b^ | 0.26 ± 0.05^c^ | 0.08 ± 0.01^d^ | ND |
| V3 | Isoamyl acetate | 0.21 ± 0.07^d^ | 0.26 ± 0.04^d^ | ND | ND | 2.87 ± 0.59^a^ | 0.90 ± 0.13^c^ | 0.87 ± 0.20^c^ | 1.81 ± 0.14^b^ | 1.34 ± 0.29^bc^ |
| V4 | Methyl formate | ND | 3.84 ± 0.76^a^ | ND | ND | ND | 2.99 ± 0.54^b^ | ND | ND | 2.42 ± 0.54^b^ |
| V5 | Benzeneacetic acid, methyl ester | 0.06 ± 0.02^b^ | 0.15 ± 0.05^b^ | 0.07 ± 0.02^b^ | 0.11 ± 0.02^b^ | 0.29 ± 0.07^a^ | 0.39 ± 0.08^a^ | 0.33 ± 0.11^a^ | 0.10 ± 0.02^b^ | 0.06 ± 0.01^b^ |
| V6 | Isoamyl isobutyrate | ND | ND | ND | ND | 0.27 ± 0.07^c^ | ND | 0.61 ± 0.15^a^ | 0.71 ± 0.14^a^ | 0.45 ± 0.08^b^ |
| V7 | 3-Methylbutyl 2-methylbutanoate | ND | ND | 0.02 ± 0.01^c^ | ND | 0.43 ± 0.11^b^ | 0.11 ± 0.01^c^ | 0.45 ± 0.04^ab^ | 0.55 ± 0.05^a^ | 0.42 ± 0.09^b^ |
| V8 | Isoamyl isovalerate | ND | ND | ND | ND | 0.20 ± 0.04^b^ | 0.12 ± 0.02^c^ | 0.47 ± 0.06^a^ | 0.24 ± 0.04^b^ | 0.24 ± 0.03^b^ |
| V9 | Ethyl isobutyrate | 1.00 ± 0.30^a^ | ND | ND | ND | 0.01 ± 0.01^b^ | ND | ND | ND | ND |
| V10 | α-​Terpinyl acetate | ND | ND | ND | ND | 0.15 ± 0.05^a^ | 0.22 ± 0.06^a^ | 0.19 ± 0.08^a^ | 0.20 ± 0.08^a^ | 0.16 ± 0.05^a^ |
| V11 | ethyl acetate | 5.03 ± 0.93^a^ | 0.16 ± 0.05^b^ | ND | ND | ND | ND | ND | 0.15 ± 0.04^b^ | ND |
| V12 | 2-Methoxyethyl acetate | ND | ND | ND | ND | ND | 0.77 ± 0.19^a^ | 0.34 ± 0.03^b^ | ND | ND |
| V13 | Linalyl acetate | ND | ND | ND | ND | ND | 0.18 ± 0.07^a^ | 0.07 ± 0.01^b^ | 0.05 ± 0.02^b^ | ND |
| V14 | Diisobutyl phthalate | 0.15 ± 0.08^a^ | 0.07 ± 0.01^b^ | 0.07 ± 0.07^b^ | 0.08 ± 0.03^ab^ | ND | ND | ND | ND | ND |
|  | **Acids (16)** |  |  |  |  |  |  |  |  |  |
| V15 | Nonanoic acid | 0.67 ± 0.04^a^ | 0.54 ± 0.11^a^ | 0.20 ± 0.18^bc^ | 0.23 ± 0.02^b^ | 0.10 ± 0.03^bcd^ | 0.08 ± 0.02^cd^ | 0.02 ± 0.01^d^ | 0.10 ± 0.02^bcd^ | 0.10 ± 0.04^bcd^ |
| V16 | 3-Methylbutanoic acid | 0.46 ± 0.16^b^ | ND | ND | ND | 3.10 ± 0.62^a^ | 3.19 ± 0.95^a^ | 2.66 ± 1.12^a^ | 1.15 ± 0.34^b^ | 0.14 ± 0.03^b^ |
| V17 | Butanoic acid | 0.06 ± 0.03^cd^ | 0.23 ± 0.01^ab^ | ND | 0.03 ± 0.01^d^ | 0.07 ± 0.04^cd^ | 0.16 ± 0.01^bc^ | ND | 0.34 ± 0.18^a^ | 0.25 ± 0.04^ab^ |
| V18 | Pentanoic acid, 4-methyl- | 0.03 ± 0.01^d^ | 0.32 ± 0.12^b^ | ND | 0.18 ± 0.01^c^ | 0.19 ± 0.09^c^ | 0.22 ± 0.02^bc^ | 0.14 ± 0.01^c^ | 0.59 ± 0.07^a^ | 0.68 ± 0.02^a^ |
| V19 | Acetic acid | ND | 5.35 ± 0.02^a^ | 3.10 ± 2.04^b^ | 1.18 ± 0.98^c^ | 0.73 ± 0.11^c^ | 0.38 ± 0.03^c^ | 0.40 ± 0.08^c^ | ND | ND |
| V20 | 2-Methylbutyric acid | 0.46 ± 0.01^e^ | 3.78 ± 0.20^b^ | 1.72 ± 0.95^d^ | 3.42 ± 1.09^bc^ | 3.11 ± 0.51^bc^ | 2.92 ± 0.35^bc^ | 2.62 ± 0.11^cd^ | ND | 7.65 ± 0.26^a^ |
| V21 | Benzeneacetic acid | 0.03 ± 0.01^c^ | 0.04 ± 0.02^c^ | ND | 0.07 ± 0.01^c^ | 0.31 ± 0.10^b^ | 0.28 ± 0.02^b^ | 0.13 ± 0.06^c^ | 0.69 ± 0.08^a^ | 0.68 ± 0.07^a^ |
| V22 | Isobutyric acid | ND | ND | 0.94 ± 0.20^bc^ | 2.33 ± 0.47^a^ | ND | 1.10 ± 0.10^b^ | ND | 2.25 ± 0.61^a^ | 0.46 ± 0.04^c^ |
| V23 | (R)-(-)-4-Methylhexanoic acid | ND | 0.26 ± 0.04^b^ | 0.04 ± 0.01^c^ | 0.06 ± 0.01^c^ | 0.29 ± 0.05^b^ | 0.51 ± 0.17^a^ | 0.41 ± 0.09^ab^ | 0.50 ± 0.09^a^ | 0.47 ± 0.03^a^ |
| V24 | Propanoic acid | ND | 0.47 ± 0.02^a^ | ND | ND | 0.05 ± 0.01^c^ | 0.04 ± 0.01^c^ | ND | 0.37 ± 0.04^b^ | 0.35 ± 0.05^b^ |
| V25 | 3-(Methylthio) propionic acid | ND | ND | ND | ND | 0.05 ± 0.02^b^ | 0.04 ± 0.02^b^ | 0.03 ± 0.01^b^ | 0.15 ± 0.02^a^ | 0.18 ± 0.07^a^ |
| V26 | Benzoic acid | 0.05 ± 0.01^c^ | ND | 0.03 ± 0.01^c^ | 0.05 ± 0.02^c^ | 0.21 ± 0.07^a^ | 0.05 ± 0.01^c^ | 0.04 ± 0.01^c^ | 0.12 ± 0.01^b^ | 0.10 ± 0.01^b^ |
| V27 | Pentanoic acid | 0.04 ± 0.01^c^ | ND | ND | ND | 0.08 ± 0.01^c^ | 0.48 ± 0.08^a^ | 0.35 ± 0.04^b^ | 0.31 ± 0.10^b^ | 0.02 ± 0.01^c^ |
| V28 | stearic acid | 0.28 ± 0.10^a^ | 0.11 ± 0.03^b^ | 0.13 ± 0.03^b^ | 0.15 ± 0.06^b^ | ND | ND | ND | ND | ND |
| V29 | Heptanoic acid | 0.11 ± 0.06^ab^ | 0.05 ± 0.01^b^ | 0.06 ± 0.01^b^ | 0.13 ± 0.08^a^ | ND | ND | ND | ND | ND |
|  | **Aldehydes (3)** |  |  |  |  |  |  |  |  |  |
| V30 | Isovaleraldehyde | ND | 0.15 ± 0.01^c^ | 0.45 ± 0.18^b^ | 0.32 ± 0.03^bc^ | ND | ND | 1.52 ± 0.21^a^ | ND | ND |
| V31 | Acetaldehyde | 0.04 ± 0.01^c^ | 0.27 ± 0.03^b^ | ND | ND | ND | ND | ND | 0.92 ± 0.10^a^ | ND |
| V32 | Benzaldehyde | 0.04 ± 0.01^c^ | 0.92 ± 0.11^b^ | 2.09 ± 0.95^a^ | 2.88 ± 0.81^a^ | 0.91 ± 0.09^b^ | 0.90 ± 0.10^b^ | 0.72 ± 0.09^bc^ | 0.26 ± 0.06^bc^ | 0.38 ± 0.04^bc^ |
|  | **Ketones（27）** |  |  |  |  |  |  |  |  |  |
| V33 | 2-Heptanone | 0.40 ± 0.01^b^ | 0.38 ± 0.03^b^ | 0.18 ± 0.02^cd^ | 0.26 ± 0.05^c^ | 0.23 ± 0.05^c^ | 0.44 ± 0.14^b^ | 0.64 ± 0.03^a^ | 0.08 ± 0.02^de^ | 0.04 ± 0.01^e^ |
| V34 | 3-Octanone | 2.87 ± 0.03^a^ | 0.94 ± 0.26^c^ | ND | 2.73 ± 0.66^a^ | 1.42 ± 0.25^bc^ | ND | 1.63 ± 0.57^b^ | 1.10 ± 0.08^bc^ | 0.96 ± 0.05^c^ |
| V35 | 2,3-Butanedione | ND | 0.88 ± 0.03^a^ | 0.81 ± 0.12^a^ | 0.36 ± 0.15^c^ | 0.10 ± 0.03^d^ | ND | 0.09 ± 0.01^d^ | 0.60 ± 0.15^b^ | 0.73 ± 0.05^ab^ |
| V36 | Acetoin | 6.83 ± 0.70^a^ | 2.92 ± 0.40^b^ | 1.71 ± 0.07^c^ | 0.56 ± 0.05^d^ | 0.46 ± 0.07^d^ | ND | ND | 1.31 ± 0.44^c^ | 0.21 ± 0.01^d^ |
| V37 | 2-Tetradecanone | ND | 0.16 ± 0.06^e^ | 1.38 ± 0.02^b^ | 1.91 ± 0.09^a^ | 0.09 ± 0.03^f^ | 0.52 ± 0.06^c^ | 0.40 ± 0.09^d^ | 0.02 ± 0.01^ef^ | ND |
| V38 | Acetyl valeryl | 0.06 ± 0.05^b^ | 0.39 ± 0.12^b^ | 1.72 ± 0.96^a^ | ND | 1.63 ± 0.62^a^ | 0.23 ± 0.05^b^ | 1.43 ± 0.39^a^ | 1.85 ± 0.13^a^ | 1.39 ± 0.35^a^ |
| V39 | 2-Hexadecanone | ND | 0.06 ± 0.03^c^ | 0.25 ± 0.13^b^ | 0.55 ± 0.09^a^ | 0.09 ± 0.02^c^ | 0.11 ± 0.02^c^ | 0.08 ± 0.02^c^ | ND | ND |
| V40 | 2-Nonanone | ND | 0.04 ± 0.02^d^ | 0.27 ± 0.04^c^ | 0.80 ± 0.06^a^ | 0.44 ± 0.11^b^ | 0.37 ± 0.13^bc^ | 0.14 ± 0.04^d^ | ND | ND |
| V41 | 2-Pentadecanone | 0.07 ± 0.04^d^ | 0.05 ± 0.01^d^ | 0.47 ± 0.06^b^ | 0.93 ± 0.17^a^ | 0.11 ± 0.01^d^ | 0.26 ± 0.04^c^ | 0.34 ± 0.06^c^ | 0.04 ± 0.01^d^ | 0.01 ± 0.01^d^ |
| V42 | 2-Heptanone, 6-methyl- | ND | 0.07 ± 0.02^cd^ | 0.32 ± 0.11^a^ | 0.25 ± 0.12^ab^ | 0.19 ± 0.07^abc^ | 0.23 ± 0.09^ab^ | 0.16 ± 0.04^bcd^ | 0.03 ± 0.01^d^ | ND |
| V43 | 2-Tridecanone | 0.04 ± 0.02^e^ | ND | 0.15 ± 0.02^d^ | 0.63 ± 0.02^a^ | 0.26 ± 0.07^c^ | 0.42 ± 0.04^b^ | 0.05 ± 0.01^e^ | ND | ND |
| V44 | 2-Butanone | ND | 0.05 ± 0.03^c^ | ND | 2.59 ± 0.98^a^ | 0.73 ± 0.08^b^ | 0.45 ± 0.10^bc^ | 0.57 ± 0.21^bc^ | ND | ND |
| V45 | 3-Hexanone, 4-methyl- | ND | ND | ND | 0.25 ± 0.08^b^ | 1.52 ± 0.31^a^ | 1.43 ± 0.55^a^ | ND | ND | ND |
| V46 | Acetone | ND | 1.69 ± 0.34^a^ | 0.36 ± 0.06^c^ | 0.48 ± 0.06^c^ | 1.42 ± 0.22^a^ | ND | 0.85 ± 0.08^b^ | ND | ND |
| V47 | Fenchone | ND | ND | ND | ND | ND | 0.27 ± 0.03^b^ | 0.37 ± 0.09^ab^ | 0.42 ± 0.09^a^ | 0.30 ± 0.09^b^ |
| V48 | 2-Acetoxy-3-butanone | 1.33 ± 0.27^a^ | ND | ND | ND | ND | ND | ND | 0.25 ± 0.12^b^ | 0.02 ± 0.01^c^ |
| V49 | Piperitone | ND | ND | ND | ND | 0.07 ± 0.01^c^ | 0.35 ± 0.04^a^ | 0.17 ± 0.07^b^ | 0.15 ± 0.05^b^ | 0.13 ± 0.02^bc^ |
| V50 | 2-Piperidinone | ND | ND | 0.02 ± 0.01^d^ | 0.05 ± 0.01^cd^ | 0.23 ± 0.09^a^ | 0.17 ± 0.03^ab^ | 0.18 ± 0.05^ab^ | 0.12 ± 0.07^bc^ | 0.11 ± 0.04^bcd^ |
| V51 | Acetophenone | 0.06 ± 0.02^c^ | 0.05 ± 0.01^c^ | ND | ND | 0.18 ± 0.05^b^ | 0.10 ± 0.01^c^ | 0.31 ± 0.10^a^ | 0.05 ± 0.02^c^ | 0.04 ± 0.01^c^ |
| V52 | 4-Octanone, 5-hydroxy-2,7-dimethyl- | ND | ND | ND | ND | ND | 0.14 ± 0.04^b^ | 0.35 ± 0.04^a^ | ND | ND |
| V53 | 2-Decanone | ND | ND | ND | 1.15 ± 0.04^b^ | ND | 0.23 ± 0.05^c^ | 1.41 ± 0.10^a^ | ND | ND |
| V54 | 2-Dodecanone | ND | ND | 0.09 ± 0.03^d^ | 0.35 ± 0.06^b^ | 0.19 ± 0.08^c^ | 0.28 ± 0.07^bc^ | 0.52 ± 0.08^a^ | ND | ND |
| V55 | 2-Hexanone | ND | 0.24 ± 0.03^c^ | ND | ND | 1.60 ± 0.07^a^ | 0.54 ± 0.04^b^ | ND | ND | ND |
| V56 | 3-Methyl-2-pentanone | ND | 0.07 ± 0.01^b^ | 0.16 ± 0.15^ab^ | 0.17 ± 0.01^a^ | ND | ND | ND | ND | ND |
| V57 | Cyclohexanone | ND | ND | 0.07 ± 0.06^b^ | 0.56 ± 0.04^a^ | ND | 0.02 ± 0.01^c^ | ND | ND | ND |
| V58 | 2,3-Pentanedione | 0.14 ± 0.09^bc^ | ND | 0.17 ± 0.03^b^ | 2.82 ± 0.18^a^ | 0.02 ± 0.01^c^ | ND | ND | ND | ND |
| V59 | 2-Hydroxy-3-pentanone | 0.97 ± 0.16^a^ | 0.40 ± 0.18^b^ | 0.07 ± 0.02^c^ | 0.12 ± 0.01^c^ | 0.11 ± 0.01^c^ | ND | 0.03 ± 0.01^c^ | 0.03 ± 0.01^c^ | 0.03 ± 0.01^c^ |
| V60 | 1-acetoxy-3-(4-acetoxy-3-methoxy-phenyl)-acetone | 1.26 ± 0.36^b^ | 0.56 ± 0.11^c^ | ND | ND | 1.34 ± 0.15^b^ | 1.11 ± 0.11^b^ | ND | ND | 1.87 ± 0.05^a^ |
|  | **Alcohols (18)** |  |  |  |  |  |  |  |  |  |
| V61 | 3-Methyl-1-butanol | 1.99 ± 0.12^cd^ | 1.96 ± 0.49^cd^ | 3.73 ± 0.29^ab^ | 2.75 ± 0.79^bcd^ | 4.84 ± 0.73^a^ | 2.89 ± 0.07^bcd^ | 2.99 ± 0.22^bc^ | 2.18 ± 0.91^cd^ | 1.81 ± 0.80^d^ |
| V62 | 1-Hexanol | 1.16 ± 0.43^a^ | 0.30 ± 0.12^b^ | ND | 0.02 ± 0.01^b^ | 0.08 ± 0.01^b^ | ND | 0.06 ± 0.01^b^ | 0.04 ± 0.01^b^ | 0.02 ± 0.01^b^ |
| V63 | 1-Propanol, 2-methyl- | 0.43 ± 0.02^b^ | 0.02 ± 0.01^d^ | 0.89 ± 0.17^a^ | 0.09 ± 0.06^cd^ | 0.52 ± 0.04^b^ | 0.10 ± 0.01^cd^ | 0.20 ± 0.01^c^ | ND | ND |
| V64 | 1-Octen-3-ol | 5.62 ± 0.90^a^ | 1.95 ± 0.08^b^ | 1.02 ± 0.03^c^ | 0.39 ± 0.01^d^ | 0.85 ± 0.07^cd^ | ND | ND | 0.75 ± 0.12^cd^ | 0.39 ± 0.08^d^ |
| V65 | Benzyl alcohol | 0.14 ± 0.04^cd^ | 0.51 ± 0.10^b^ | 0.82 ± 0.17^a^ | 0.17 ± 0.07^cd^ | 0.68 ± 0.04^a^ | 0.17 ± 0.01^cd^ | 0.03 ± 0.01^d^ | 0.28 ± 0.07^c^ | 0.14 ± 0.04^cd^ |
| V66 | 2,3-Butanediol | 7.66 ± 0.11^a^ | 3.04 ± 0.97^b^ | ND | ND | ND | ND | ND | ND | 1.92 ± 0.09^c^ |
| V67 | Phenylethyl Alcohol | 1.65 ± 0.05^b^ | 1.41 ± 0.05^bc^ | 0.86 ± 0.24^d^ | 0.26 ± 0.06^e^ | 1.65 ± 0.13^b^ | 1.45 ± 0.18^bc^ | 2.01 ± 0.39^a^ | 1.21 ± 0.12^cd^ | 1.21 ± 0.09^cd^ |
| V68 | 3-Octanol | 1.04 ± 0.07^a^ | 0.21 ± 0.04^d^ | ND | ND | ND | 0.37 ± 0.13^c^ | ND | 0.53 ± 0.03^b^ | 0.27 ± 0.07^cd^ |
| V69 | 4-Heptanol, 3,5-dimethyl- | ND | 1.21 ± 0.10^a^ | 0.32 ± 0.05^b^ | ND | ND | ND | ND | ND | 0.25 ± 0.04^b^ |
| V70 | 3-Pentanol, 2,4-dimethyl- | ND | ND | 0.52 ± 0.18^a^ | 0.11 ± 0.10^b^ | 0.20 ± 0.05^b^ | ND | 0.09 ± 0.01^b^ | 0.15 ± 0.05^b^ | 0.14 ± 0.03^b^ |
| V71 | Linalool | ND | ND | ND | ND | 0.38 ± 0.08^a^ | 0.41 ± 0.12^a^ | 0.38 ± 0.06^a^ | 0.45 ± 0.07^a^ | 0.41 ± 0.01^a^ |
| V72 | 1-Phenyl-2-propanol | ND | ND | ND | ND | 0.32 ± 0.11^b^ | 0.27 ± 0.06^b^ | 0.56 ± 0.14^a^ | ND | ND |
| V73 | 4-thujanol | ND | ND | ND | ND | 0.34 ± 0.04^bc^ | 0.61 ± 0.06^a^ | 0.63 ± 0.18^a^ | 0.39 ± 0.08^b^ | 0.21 ± 0.03^c^ |
| V74 | (+)-alpha-terpineol | ND | ND | ND | ND | 0.77 ± 0.06^a^ | ND | 0.43 ± 0.06^b^ | 0.38 ± 0.09^b^ | ND |
| V75 | Terpinen-4-ol | ND | ND | ND | ND | ND | ND | 0.78 ± 0.07^a^ | 0.44 ± 0.06^b^ | 0.33 ± 0.02^c^ |
| V76 | (2R,3R)-2,3-Butanediol | ND | ND | ND | ND | ND | ND | ND | 3.39 ± 0.53^a^ | 0.61 ± 0.05^b^ |
| V77 | Eucalyptol | ND | ND | ND | ND | 0.70 ± 0.13^c^ | 3.45 ± 0.56^a^ | 0.55 ± 0.05^c^ | ND | 1.82 ± 0.56^b^ |
| V78 | 2-Isopropyl-5-methyl-1-heptanol | ND | ND | 0.36 ± 0.17^b^ | 0.68 ± 0.05^a^ | ND | ND | ND | ND | ND |
|  | **Pyrazines (9)** |  |  |  |  |  |  |  |  |  |
| V79 | 2,3,5,6-Tetramethylpyrazine | ND | 0.25 ± 0.15^d^ | 2.43 ± 0.08^b^ | 2.43 ± 0.67^b^ | 0.33 ± 0.07^d^ | 0.57 ± 0.03^d^ | 1.83 ± 0.14^c^ | 1.40 ± 0.36^c^ | 3.42 ± 0.29^a^ |
| V80 | 2,3,5-Trimethylpyrazine | 0.08 ± 0.02^e^ | 1.18 ± 0.04^de^ | 2.44 ± 0.98^cb^ | 2.87 ± 0.13^b^ | 1.58 ± 0.15^cd^ | 1.75 ± 0.18^bcd^ | 4.47 ± 1.08^a^ | 1.86 ± 0.77^bcd^ | ND |
| V81 | 2,5-Dimethylpyrazine | 0.06 ± 0.01^d^ | 0.25 ± 0.06^d^ | 1.92 ± 0.08^b^ | 2.63 ± 0.55^a^ | 1.97 ± 0.07^b^ | 2.03 ± 0.69^ab^ | 1.57 ± 0.11^bc^ | 1.40 ± 0.35^bc^ | 0.92 ± 0.05^c^ |
| V82 | 2-Ethyl-3,5,6-trimethylpyrazine | ND | 0.38 ± 0.02^b^ | 1.53 ± 0.89^a^ | 1.67 ± 0.18^a^ | 0.13 ± 0.04^b^ | 0.15 ± 0.05^b^ | 1.60 ± 0.31^a^ | 0.77 ± 0.07^b^ | 0.51 ± 0.11^b^ |
| V83 | 2-Ethyl-3,5-dimethylpyrazine | ND | 0.39 ± 0.17^c^ | 1.60 ± 0.04^b^ | 2.41 ± 0.84^a^ | 0.24 ± 0.05^c^ | 0.76 ± 0.04^c^ | 0.74 ± 0.04^c^ | 0.27 ± 0.06^c^ | 0.20 ± 0.09^c^ |
| V84 | Pyrazine, 2,3-dimethyl- | ND | ND | 0.45 ± 0.16^a^ | ND | 0.06 ± 0.01^c^ | 0.08 ± 0.01^c^ | 0.30 ± 0.08^b^ | 0.16 ± 0.05^bc^ | 0.27 ± 0.07^b^ |
| V85 | Pyrazine, 2,6-dimethyl- | ND | 1.62 ± 0.37^b^ | 2.08 ± 0.12^a^ | 0.36 ± 0.01^cd^ | 0.31 ± 0.06^cd^ | 0.43 ± 0.05^c^ | 0.35 ± 0.04^cd^ | 0.21 ± 0.07^cd^ | 0.14 ± 0.03^d^ |
| V86 | Pyrazine, 2-ethyl-6-methyl- | ND | ND | ND | 0.09 ± 0.02^b^ | 0.09 ± 0.01^b^ | 0.46 ± 0.05^b^ | 1.02 ± 0.69^a^ | 0.08 ± 0.02^b^ | 0.09 ± 0.02^b^ |
| V87 | Pyrazine, methyl- | ND | ND | ND | ND | ND | 0.19 ± 0.06^a^ | 0.12 ± 0.07^b^ | 0.05 ± 0.01^c^ | ND |
|  | **Alkanes（17）** |  |  |  |  |  |  |  |  |  |
| V88 | Decane | 0.40 ± 0.19^cd^ | 0.50 ± 0.12^cd^ | 1.09 ± 0.01^a^ | 1.07 ± 0.04^a^ | 0.60 ± 0.08^bc^ | ND | 0.51 ± 0.06^cd^ | 0.31 ± 0.27^d^ | 0.81 ± 0.08^b^ |
| V89 | Dodecane | 0.19 ± 0.10^cd^ | 0.31 ± 0.01^cd^ | 0.75 ± 0.17^b^ | 1.32 ± 0.51^a^ | 0.49 ± 0.08^bc^ | ND | 0.03 ± 0.01^d^ | 0.36 ± 0.05^cd^ | 0.02 ± 0.01^d^ |
| V90 | Tridecane | ND | 0.31 ± 0.15^b^ | 0.11 ± 0.01^de^ | ND | 0.23 ± 0.10^bcd^ | 0.17 ± 0.02^cd^ | 0.26 ± 0.05^bc^ | 0.02 ± 0.01^e^ | 0.50 ± 0.02^a^ |
| V91 | Undecane | 0.06 ± 0.02^d^ | 0.07 ± 0.02^d^ | 0.11 ± 0.03^cd^ | 0.03 ± 0.02^d^ | 0.31 ± 0.05^b^ | 0.07 ± 0.02^d^ | 0.27 ± 0.05^b^ | 0.18 ± 0.05^c^ | 1.54 ± 0.04^a^ |
| V92 | Hexadecane | 0.08 ± 0.03^d^ | 0.22 ± 0.01^c^ | 0.05 ± 0.01^de^ | 1.47 ± 0.01^a^ | 0.35 ± 0.04^b^ | 0.18 ± 0.05^c^ | 0.03 ± 0.01^e^ | ND | 0.04 ± 0.01^de^ |
| V93 | β-myrcene | ND | ND | ND | ND | 0.52 ± 0.06^bc^ | 0.44 ± 0.03^c^ | 0.70 ± 0.08^a^ | 0.56 ± 0.06^b^ | 0.54 ± 0.04^b^ |
| V94 | D-limonene | ND | ND | ND | 0.03 ± 0.01^c^ | 1.33 ± 0.21^ab^ | 0.99 ± 0.19^b^ | 1.84 ± 0.75^a^ | 1.11 ± 0.10^b^ | 0.94 ± 0.04^b^ |
| V95 | trans-β-Ocimene | ND | ND | ND | ND | 0.20 ± 0.05^ab^ | 0.12 ± 0.07^b^ | 0.23 ± 0.06^a^ | 0.29 ± 0.08^a^ | 0.29 ± 0.08^a^ |
| V96 | γ-Terpinene | ND | ND | ND | ND | 0.42 ± 0.08^c^ | 0.73 ± 0.06^a^ | 0.53 ± 0.06^b^ | 0.65 ± 0.08^a^ | 0.38 ± 0.06^c^ |
| V97 | Anethole | ND | ND | ND | ND | 0.87 ± 0.08^b^ | 0.89 ± 0.01^b^ | 1.46 ± 0.45^a^ | 1.51 ± 0.11^a^ | ND |
| V98 | Nonane, 2,6-dimethyl- | ND | ND | 0.14 ± 0.03^d^ | ND | ND | ND | 0.34 ± 0.04^b^ | 0.21 ± 0.06^c^ | 0.55 ± 0.05^a^ |
| V99 | Pentadecane | 0.08 ± 0.01^c^ | ND | 0.06 ± 0.01^cd^ | 0.27 ± 0.03^a^ | ND | 0.02 ± 0.01^e^ | 0.02 ± 0.01^de^ | 0.15 ± 0.04^b^ | ND |
| V100 | Dodecane, 2,7,10-trimethyl- | ND | ND | ND | ND | ND | ND | 0.35 ± 0.05^a^ | 0.04 ± 0.01^b^ | 0.03 ± 0.01^b^ |
| V101 | Dodecane, 4,6-dimethyl- | ND | ND | 0.03 ± 0.01^b^ | ND | 0.25 ± 0.07^a^ | 0.26 ± 0.04^a^ | 0.25 ± 0.05^a^ | 0.09 ± 0.01^b^ | ND |
| V102 | 3-Ethyl-3-methylheptane | ND | ND | 0.04 ± 0.01^b^ | 0.16 ± 0.02^a^ | 0.18 ± 0.07^a^ | 0.03 ± 0.01^b^ | 0.15 ± 0.04^a^ | ND | ND |
| V103 | Sabinene | ND | ND | ND | ND | 0.10 ± 0.02^b^ | 0.15 ± 0.04^b^ | 0.23 ± 0.05^a^ | 0.12 ± 0.06^b^ | 0.11 ± 0.01^b^ |
| V104 | beta-elemene | ND | ND | ND | ND | 0.17 ± 0.05^b^ | 0.31 ± 0.09^a^ | 0.16 ± 0.06^b^ | 0.07 ± 0.02^c^ | ND |
|  | **Phenols（8）** |  |  |  |  |  |  |  |  |  |
| V105 | Phenol | 0.20 ± 0.09^e^ | 0.45 ± 0.02^d^ | 0.14 ± 0.05^e^ | 0.03 ± 0.01^f^ | 0.86 ± 0.05^a^ | 0.58 ± 0.04^c^ | 0.56 ± 0.05^cd^ | 0.70 ± 0.09^b^ | 0.65 ± 0.03^bc^ |
| V106 | Maltol | 3.22 ± 0.22^a^ | 0.97 ± 0.32^b^ | 0.39 ± 0.08^de^ | 0.68 ± 0.22^bcd^ | 0.44 ± 0.04^de^ | 0.34 ± 0.04^de^ | 0.28 ± 0.08^e^ | 0.82 ± 0.09^bc^ | 0.54 ± 0.04^cde^ |
| V107 | 2-Methoxy-4-vinylphenol | 0.06 ± 0.02^de^ | 0.22 ± 0.02^b^ | 0.03 ± 0.01^e^ | 0.05 ± 0.01^de^ | 0.26 ± 0.05^ab^ | 0.29 ± 0.04^a^ | 0.11 ± 0.01^c^ | ND | 0.09 ± 0.01^cd^ |
| V108 | Guaiacol | 0.09 ± 0.06^f^ | 0.49 ± 0.02^cd^ | 0.54 ± 0.02^bc^ | 1.13 ± 0.04^a^ | 0.49 ± 0.11^cd^ | 0.65 ± 0.05^b^ | 0.42 ± 0.07^d^ | 0.21 ± 0.08^e^ | 0.15 ± 0.02^ef^ |
| V109 | Phenol, 4-ethyl-2-methoxy- | ND | 0.06 ± 0.01^c^ | 0.03 ± 0.02^c^ | 0.03 ± 0.01^c^ | 0.16 ± 0.05^b^ | 0.23 ± 0.02^b^ | 0.07 ± 0.03^c^ | 0.72 ± 0.07^a^ | 0.23 ± 0.04^b^ |
| V110 | Phenol, 4-ethyl- | 0.03 ± 0.01^c^ | 0.02 ± 0.01^c^ | ND | 0.04 ± 0.01^c^ | ND | ND | ND | 0.97 ± 0.06^a^ | 0.45 ± 0.04^b^ |
| V111 | 2,4-Di-tert-butylphenol | 0.11 ± 0.01^bc^ | 0.02 ± 0.01^d^ | ND | 0.04 ± 0.01^d^ | 0.23 ± 0.08^a^ | 0.16 ± 0.05^b^ | 0.11 ± 0.02^bc^ | 0.08 ± 0.02^cd^ | 0.05 ± 0.01^cd^ |
| V112 | tert-Butylhydroquinone | ND | ND | ND | ND | 1.81 ± 0.11^a^ | 0.03 ± 0.01^b^ | 0.03 ± 0.01^b^ | 0.02 ± 0.01^b^ | 0.02 ± 0.01^b^ |
|  | **Others (22)** |  |  |  |  |  |  |  |  |  |
| V113 | N,N-dimethylmethanamine | 0.64 ± 0.18^b^ | 0.11 ± 0.01^c^ | 0.04 ± 0.01^c^ | 1.51 ± 0.01^a^ | 0.12 ± 0.01^c^ | ND | ND | ND | 0.05 ± 0.01^c^ |
| V114 | Toluene | 0.28 ± 0.03^cd^ | ND | 0.07 ± 0.02^e^ | 0.17 ± 0.03^de^ | 0.48 ± 0.05^b^ | 0.26 ± 0.02^cd^ | 0.42 ± 0.08^bc^ | 0.21 ± 0.10^de^ | 0.78 ± 0.21^a^ |
| V115 | Disulfide, dimethyl | ND | ND | ND | 2.58 ± 0.08^a^ | 1.51 ± 0.07^b^ | 1.43 ± 0.13^b^ | 0.55 ± 0.04^c^ | 0.02 ± 0.01^d^ | 0.02 ± 0.01^d^ |
| V116 | Furan, 2-pentyl- | 0.03 ± 0.02^c^ | 0.41 ± 0.12^a^ | 0.11 ± 0.01^bc^ | 0.14 ± 0.03^b^ | 0.13 ± 0.06^b^ | 0.04 ± 0.02^c^ | 0.04 ± 0.01^bc^ | 0.09 ± 0.01^bc^ | 0.08 ± 0.01^bc^ |
| V117 | Dimethyl trisulfide | ND | 0.15 ± 0.01^d^ | 0.60 ± 0.07^b^ | 0.96 ± 0.02^a^ | 0.31 ± 0.06^bcd^ | 0.46 ± 0.03^c^ | 0.54 ± 0.04^b^ | 0.05 ± 0.01^e^ | 0.09 ± 0.01^de^ |
| V118 | Benzene, 1,3-bis(1,1-dimethylethyl)- | 1.50 ± 0.01^a^ | 0.84 ± 0.19^c^ | 1.06 ± 0.03^b^ | 1.47 ± 0.03^a^ | 0.80 ± 0.08^cd^ | 0.69 ± 0.01^cd^ | 0.74 ± 0.05^cd^ | 0.38 ± 0.06^e^ | 0.64 ± 0.08^d^ |
| V119 | Estragole | ND | ND | ND | ND | 1.82 ± 0.31^a^ | 1.62 ± 0.30^ab^ | 1.37 ± 0.19^b^ | 1.85 ± 0.14^a^ | 0.42 ± 0.04^c^ |
| V120 | Benzofuran, 2,3-dihydro- | 0.13 ± 0.11^e^ | 0.46 ± 0.03^bc^ | 0.18 ± 0.01^de^ | 0.24 ± 0.01^d^ | 0.56 ± 0.05^b^ | 0.45 ± 0.02^bc^ | 0.68 ± 0.07^a^ | 0.42 ± 0.03^c^ | 0.47 ± 0.07^bc^ |
| V121 | Indole | 0.02 ± 0.01^e^ | 0.02 ± 0.01^e^ | ND | ND | 0.46 ± 0.07^b^ | 1.93 ± 0.19^a^ | 0.07 ± 0.01^de^ | 0.27 ± 0.07^c^ | 0.20 ± 0.09^cd^ |
| V122 | Nitrous oxide | ND | 0.04 ± 0.01^c^ | ND | 0.75 ± 0.03^b^ | 0.04 ± 0.01^c^ | ND | 1.38 ± 0.13^a^ | ND | 0.07 ± 0.02^c^ |
| V123 | Oxazole, trimethyl- | ND | 0.07 ± 0.01^d^ | 0.48 ± 0.03^a^ | 0.43 ± 0.03^a^ | ND | 0.04 ± 0.01^d^ | 0.13 ± 0.06^bc^ | 0.17 ± 0.05^b^ | 0.09 ± 0.02^cd^ |
| V124 | Hydrazine, ethyl- | ND | ND | ND | ND | ND | ND | ND | 4.24 ± 0.88^a^ | 1.69 ± 0.21^b^ |
| V125 | Propanoic acid, anhydride | ND | ND | 2.98 ± 0.07^a^ | 0.17 ± 0.05^d^ | ND | ND | 0.20 ± 0.09^d^ | 2.18 ± 0.38^b^ | 1.22 ± 0.13^c^ |
| V126 | N, N, O-Triacetylhydroxylamine | 0.13 ± 0.07^d^ | 2.96 ± 0.07^a^ | 1.76 ± 0.08^b^ | ND | 0.04 ± 0.01^d^ | ND | ND | ND | 1.18 ± 0.23^c^ |
| V127 | Acetamide, N-(2-methylpropyl)- | ND | 0.04 ± 0.01^d^ | 0.13 ± 0.02^d^ | 0.37 ± 0.07^c^ | 0.48 ± 0.06^c^ | 1.68 ± 0.24^a^ | 0.76 ± 0.12^b^ | 0.43 ± 0.05^c^ | 0.36 ± 0.11^c^ |
| V128 | Benzene, 1-methyl-3-(1-methylethyl)- | ND | ND | ND | ND | 0.16 ± 0.05^a^ | 0.17 ± 0.07^a^ | 0.17 ± 0.07^a^ | 0.19 ± 0.05^a^ | 0.17 ± 0.07^a^ |
| V129 | Butanamide, 3-methyl- | ND | ND | 0.04 ± 0.01^e^ | 0.09 ± 0.01^cd^ | 0.10 ± 0.01^c^ | 0.18 ± 0.04^b^ | 0.26 ± 0.06^a^ | 0.05 ± 0.01^de^ | 0.05 ± 0.01^de^ |
| V130 | 1H-Pyrrole, 2-methyl- | ND | ND | ND | 0.03 ± 0.01^b^ | 0.21 ± 0.05^a^ | ND | ND | ND | ND |
| V131 | Pyridine, 2-ethyl-6-methyl- | ND | ND | 0.04 ± 0.01^d^ | 0.17 ± 0.01^a^ | 0.06 ± 0.01^bc^ | 0.06 ± 0.01^c^ | 0.08 ± 0.01^b^ | ND | ND |
| V132 | Butanenitrile | ND | ND | 3.71 ± 0.09^a^ | 2.77 ± 0.06^b^ | ND | ND | 0.04 ± 0.01^c^ | ND | ND |
| V133 | 2-Propen-1-amine | ND | 0.26 ± 0.08^c^ | 0.66 ± 0.04^a^ | 0.51 ± 0.08^b^ | ND | ND | ND | ND | ND |
| V134 | 1H-1,2,4-Triazole | ND | 3.68 ± 0.19^a^ | 0.04 ± 0.01^b^ | ND | ND | ND | ND | ND | ND |

Note: The values are expressed as mean ± standard deviation; ‘ND’ represented the volatile compounds was not identified in Douchiba; ^a-f^ Values in a column with different lowercase letters are significantly differ.

Table S2

The main aroma components (ROAV≥1) in the fermentation process of Douchiba.

| Compounds | Threshold (μg /kg) | Flavor description | ROAV | | | | | | | | |
| --- | --- | --- | --- | --- | --- | --- | --- | --- | --- | --- | --- |
|  |  |  | D1 | D2 | D3 | D4 | D5 | D6 | D7 | D8 | D9 |
| Butanoic acid, 3-methyl-, ethyl ester | 0.20 | Sweet, pineapple | 68.40 | - | - | - | 9.03 | 4.87 | 5.98 | 14.37 | 4.81 |
| Isoamyl acetate | 2.00 | Banana | 1.23 | 1.55 | - | - | 16.81 | 5.26 | 5.13 | 10.64 | 7.86 |
| Ethyl acetate | 5.00 | Fruit | 11.80 | 0.38 | - | - | - | - | - | 0.34 | - |
| 3-Methylbutanoic acid | 12.00 | Medicine | 0.45 | - | - | - | 3.03 | 3.11 | 2.60 | 1.13 | 0.14 |
| Acetic acid | 13.00 | Sour | - | 4.83 | 2.79 | 1.06 | 0.66 | 0.34 | 0.36 | - | 0.00 |
| 2-Methylbutyric acid | 10.00 | Cheese | 0.54 | 4.43 | 2.01 | 4.01 | 3.65 | 3.43 | 3.07 | - | 8.98 |
| Isobutyric acid | 10.00 | Butter, cheese | - | - | 1.10 | 2.73 | - | 1.29 | - | 2.64 | 0.54 |
| Isovaleraldehyde | 1.10 | Chocolate, cocoa, fruit. | - | 1.62 | 4.82 | 3.39 | - | - | 16.22 | - | - |
| Benzaldehyde | 24.00 | Bitter almond, caramel, roasted pepper | 0.02 | 0.45 | 1.02 | 1.41 | 0.44 | 0.44 | 0.35 | 0.13 | 0.18 |
| 3-Octanone | 21.00 | Mushroom, resin, leather | 1.61 | 0.52 | - | 1.53 | 0.79 | - | 0.91 | 0.62 | 0.54 |
| Acetoin | 14.00 | Butter, cream, pepper | 5.73 | 2.44 | 1.43 | 0.47 | 0.39 | - | - | 1.10 | 0.17 |
| 2-Decanone | 3.00 | Cheese, fat, fruit | - | - | - | 4.49 | - | 0.88 | 5.53 | - | - |
| 2,3-Pentanedione | 20.00 | Buttery | 0.08 | - | 0.10 | 1.65 | 0.01 | - | - | - | - |
| 3-Methyl-1-butanol | 4.00 | Pineapple, fruit | 5.83 | 5.75 | 10.94 | 8.07 | 14.20 | 8.49 | 8.77 | 6.40 | 5.31 |
| 1-Octen-3-ol | 1.50 | Mushroom, hay | 43.93 | 15.26 | 7.99 | 3.06 | 6.64 | - | - | 5.83 | 3.03 |
| 2,3-Butanediol | 15.00 | Fat | 5.99 | 2.38 | - | - | - | - | - | - | 1.50 |
| Eucalyptol | 1.10 | Camphor, mint | - | - | - | - | 7.51 | 36.79 | 5.89 | - | 19.37 |
| 2,3,5-Trimethylpyrazine | 23.00 | Earthy, musty, dusty | 0.04 | 0.60 | 1.24 | 1.46 | 0.81 | 0.89 | 2.28 | 0.95 | - |
| 2-Ethyl-3,5-dimethylpyrazine | 8.60 | - | - | 0.53 | 2.18 | 3.29 | 0.33 | 1.04 | 1.01 | 0.37 | 0.28 |
| D-Limonene | 12.00 | Citrus, sweetness | - | - | - | 0.03 | 1.30 | 0.97 | 1.80 | 1.08 | 0.92 |
| Anethole | 15.00 | Anise | - | - | - | - | 0.68 | 0.70 | 1.14 | 1.18 | - |
| Maltol | 2.50 | Sweet, caramelized | 15.13 | 4.56 | 1.82 | 3.20 | 2.07 | 1.61 | 1.33 | 3.85 | 2.54 |
| Guaiacol | 10.00 | Spicy, smoky | 0.10 | 0.57 | 0.63 | 1.33 | 0.57 | 0.76 | 0.49 | 0.25 | 0.17 |
| N,N-dimethylmethanamine | 8.00 | Fishy, harry | 0.94 | 0.16 | 0.06 | 2.22 | 0.17 | - | - | - | 0.07 |
| Disulfide, dimethyl | 0.30 | Onion, garlic | - | - | - | 100.00 | 58.98 | 55.81 | 21.35 | 0.70 | 0.82 |
| Estragole | 16.00 | Licorice, anise | - | - | - | - | 1.33 | 1.18 | 1.01 | 1.36 | 0.31 |
| Indole | 11.00 | Floral, camphoraceous | 0.02 | 0.02 | - | - | 0.49 | 2.06 | 0.07 | 0.29 | 0.21 |
| Butanenitrile | 32.00 | - | - | - | 1.36 | 1.01 | - | - | 0.02 | - | - |

Note: “-”, not detected; Threshold was retrieved from the book named Compilations of Odour Threshold Values in Air, Water and Other media.
